# Aberrant signaling of immune cells in Sjögren’s syndrome patient subgroups upon interferon stimulation

**DOI:** 10.3389/fimmu.2022.854183

**Published:** 2022-08-22

**Authors:** Irene Sarkar, Richard Davies, Anders K. Aarebrot, Silje M. Solberg, Aleksandra Petrovic, Anagha M. Joshi, Brith Bergum, Johan G. Brun, Daniel Hammenfors, Roland Jonsson, Silke Appel

**Affiliations:** ^1^ Broegelmann Research Laboratory, Department of Clinical Science, University of Bergen, Bergen, Norway; ^2^ Department of Dermatology, Haukeland University Hospital, Bergen, Norway; ^3^ Computational Biology Unit, Department of Clinical Science, University of Bergen, Bergen, Norway; ^4^ Core Facility for Flow Cytometry, Department of Clinical Science, University of Bergen, Bergen, Norway; ^5^ Department of Rheumatology, Haukeland University Hospital, Bergen, Norway; ^6^ Department of Clinical Science, University of Bergen, Bergen, Norway

**Keywords:** mass cytometry (CyTOF), autoantibodies, Sjögren’s syndrome, type I and type II interferons, phospho-signaling, patient stratification

## Abstract

**Background:**

Primary Sjögren’s syndrome (pSS) is a systemic autoimmune disease, characterized by mononuclear cell infiltrates in the salivary and lacrimal glands, leading to glandular atrophy and dryness. Patient heterogeneity and lack of knowledge regarding its pathogenesis makes pSS a difficult disease to manage.

**Methods:**

An exploratory analysis using mass cytometry was conducted of MAPK/ERK and JAK/STAT signaling pathways in peripheral blood mononuclear cells (PBMC) from 16 female medication free pSS patients (8 anti-Sjögren’s syndrome-related antigen A negative/SSA- and 8 SSA+) and 8 female age-matched healthy donors after stimulation with interferons (IFNs).

**Results:**

We found significant differences in the frequencies of memory B cells, CD8+ T central and effector memory cells and terminally differentiated CD4+ T cells among the healthy donors and patient subgroups. In addition, we observed an upregulation of HLA-DR and CD38 in many cell subsets in the patients. Upon IFNα2b stimulation, slightly increased signaling through pSTAT1 Y701 was observed in most cell types in pSS patients compared to controls, while phosphorylation of STAT3 Y705 and STAT5 Y694 were slightly reduced. IFNγ stimulation resulted in significantly increased pSTAT1 Y701 induction in conventional dendritic cells (cDCs) and classical and non-classical monocytes in the patients. Most of the observed differences were more prominent in the SSA+ subgroup, indicating greater disease severity in them.

**Conclusions:**

Augmented activation status of certain cell types along with potentiated pSTAT1 Y701 signaling and reduced pSTAT3 Y705 and pSTAT5 Y694 induction may predispose pSS patients, especially the SSA+ subgroup, to upregulated expression of IFN-induced genes and production of autoantibodies. These patients may benefit from therapies targeting these pathways.

## Introduction

Primary Sjögren’s syndrome (pSS) is a chronic, systemic, inflammatory autoimmune disorder characterized by progressive mononuclear cell infiltration in the salivary and lacrimal glands causing glandular dysfunction. This leads to oral dryness (xerostomia) and ocular dryness (keratoconjunctivitis sicca) (1). pSS has a prevalence of approximately 0.01-3.0% in the general population and 0.05% in Norway (2–4). Hallmarks of the disease include presence of autoantibodies against ribonucleoprotein particles Sjögren’s syndrome-related antigen A (SSA/Ro) and B (SSB/La), often years before disease onset (5, 6). Most of the patients are post-menopausal women (female: male ratio 9:1) who experience a severe reduction in quality of life. Currently, the treatment for pSS is restricted to symptomatic care only. Although the mechanism of disease pathogenesis is unknown, genetic predisposition coupled with viral infections, environmental and hormonal factors are all thought to contribute to the disease etiology and progression (7). Apart from the sicca symptoms, many patients also suffer from a wide range of systemic complications, called extraglandular manifestations (EGM) (5). Such variation in disease features may indicate distinct patient subgroups, driven by separate pathophysiological mechanisms (8). Separate clusters (clusters 1-5) of pSS patients have been identified by Mariette et al., with clusters 1-3 representing patients with low disease activity and clusters 4 and 5 represented patients with moderate to high disease activity (EULAR Sjögren’s syndrome disease activity index/ESSDAI >5) (9). Patient heterogeneity may explain why biologic drugs tested in clinical trials have been unsuccessful so far (10). Thus, stratification of pSS patients will considerably help in personalizing treatment for the different patient subgroups.

Altered cell frequencies in peripheral blood mononuclear cells (PBMC) of pSS patients have been reported previously (9, 11, 12). Such changes have also been correlated to disease activity and presence of autoantibodies (9, 13). Over the last decade growing evidence has pointed towards the involvement of type I IFNs in autoimmune disorders including systemic lupus erythematosus (SLE), rheumatoid arthritis (RA) and scleroderma (14). An increased expression of type I IFN-inducible genes (ISGs), due to the dysregulation of IFN signaling pathways, called the ‘IFN signature’, has been observed in the salivary glands and peripheral blood of approximately 50% of pSS patients (15, 16) and correlates with disease severity (17, 18). Although type I IFNs were proposed to be the predominant contributors to pSS pathogenesis, recent evidence suggests a role of type II IFN, IFNγ, as well (19, 20). Both type I and II IFNs have been reported to contribute to distinct SS phenotypes and distinct biological disease activities (8, 21). Genetic association studies indicate the importance of multiple genetic loci within both type I and type II IFN pathways (16). Systemic type I and II IFN activation has also been associated with higher levels of anti-SSA and anti-SSB autoantibodies (21). In fact, Versnel and colleagues have shown that the patients can be grouped into three categories based on systemic IFN activity: IFN inactive, IFN I and IFN I + II (21). IFNs signal through a family of transcription factors called signal transducers and activators of transcription (STATs). Previous studies on peripheral blood from pSS patients have found altered basal phosphorylation levels of STAT3 and STAT5, as well as increased phosphorylation of STAT1 Y701 upon IFNα, IFNγ or IL-6 stimulation (22–24). We have also shown previously that IFNα2b stimulation of PBMC leads to significantly different phosphorylation profiles in pSS patients and healthy individuals (25). Moreover, single nucleotide polymorphisms in two genes in the IFN pathway, STAT4 and IRF5, have been strongly associated with pSS (26). Therefore, aberrant signaling potential may play a crucial role in establishing distinct molecular subtypes in these patients. However, the exact mechanism is not yet well characterized.

The aim of this study was to compare the immune-profiles of SSA+ and SSA- medication-free pSS patients with healthy controls and to explore the phospho-signaling landscape in them. We conducted an exploratory analysis of MAPK/ERK and JAK/STAT signaling pathways in PBMC stimulated with IFNα2b or IFNγ, respectively, by mass cytometry.

## Results

### SSA- and SSA+ pSS patients and healthy controls display differences in frequencies of PBMC subsets

**Figure 1:**
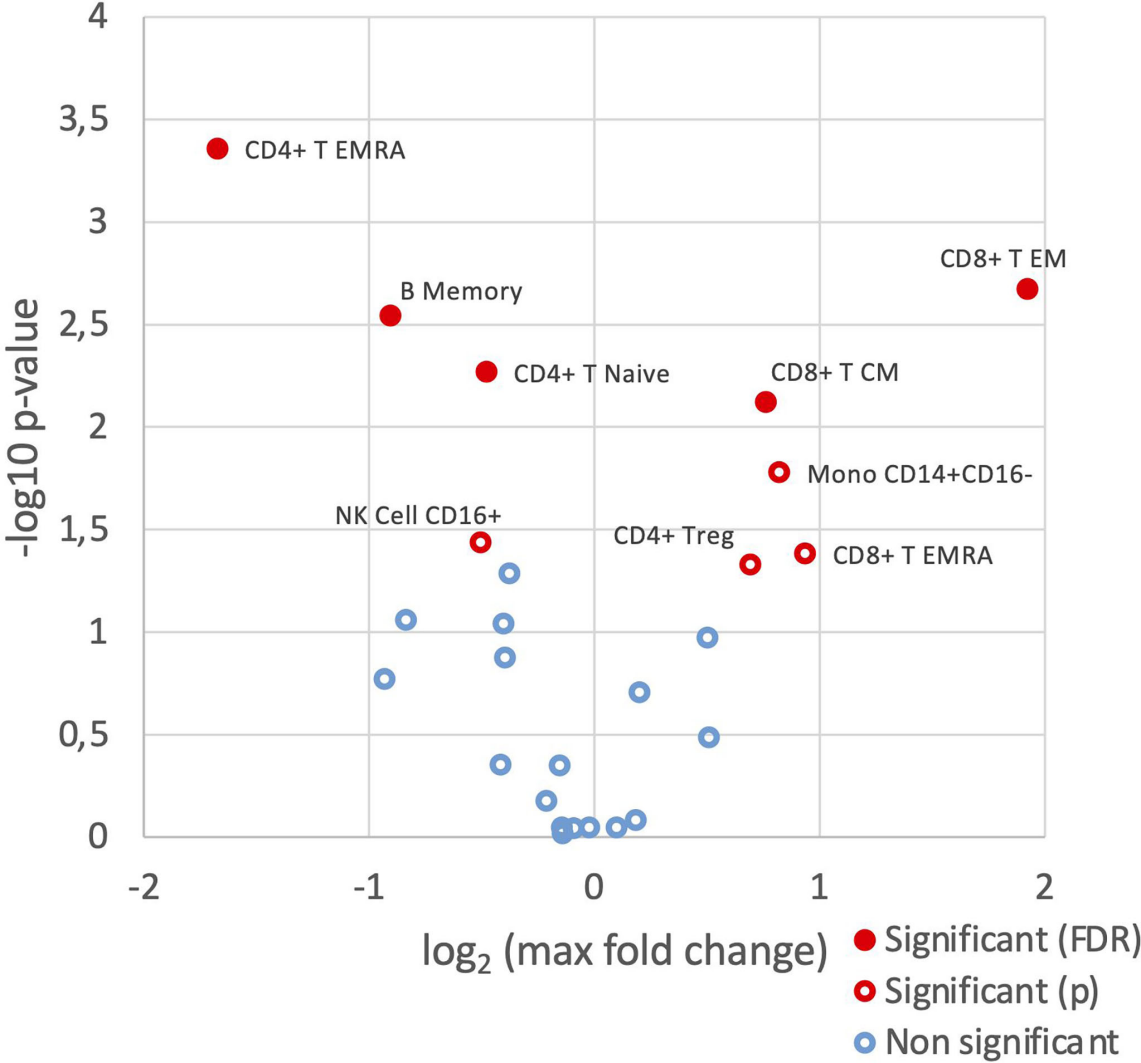
SSA- and SSA+ pSS patients and healthy controls display differences in frequencies of PBMC subsets. Immunophenotyping of PBMC was done by mass cytometry, followed by comparison of the frequencies of cellular subsets (% of CD45+ cells) identified using a hierarchical clustering approach, among healthy controls (n=8), SSA- pSS patients (n=8) and SSA+ pSS patients (n=8). Differential abundance analysis was done using the edgeR R package, and pairwise comparisons were made between the 3 groups. Overview of differential abundance of cellular subsets with cell subsets with altered frequencies between the groups highlighted in red (p and FDR ≤ 0.05), with closed symbols indicating differences which were still significant after correcting for multiple testing and max fold change between groups given on the X-axis.

Although cell frequencies of the parent populations did not vary significantly across the three groups, differences were observed in the frequencies of the various cell subsets between HC, SSA- and SSA+ pSS patients (**Figure 1** and **Supplementary Table S1**). Memory B cell frequency was significantly reduced in SSA+ patients compared to HC (false discovery rate/FDR = 0.013) as well as between SSA- and SSA+ patients (FDR = 0.034). The frequency of naïve B cells was slightly increased in the pSS patient subgroups. A decrease in the frequency of CD8+ T central memory (CM) cells was seen in the SSA+ patients compared to HC and SSA- patients (that reached a significance value of FDR = 0.032 only in the groupwise comparison but not in the pairwise comparisons). An increase was seen in CD8+ T effector memory (EM) cells in both patient subgroups compared to HC, that reached significance between HC and SSA- patients (FDR = 0.015) and an increasing tendency was seen for the terminally differentiated CD8+ T (EMRA) cells. A slight decrease was seen in the CD4+ T EM cell frequency, being most prominent on comparing HC and SSA+ patients. A significant decrease in CD4+ T EMRA frequency was found on comparing HC and SSA+ pSS patients (FDR = 0.013). A progressively decreasing frequency was also observed in the CD56+CD16+ NK cell subset in the patient subgroups. Amongst the myeloid cells, cDCs and pDCs had slightly decreased frequencies, while classical monocytes were slightly increased in both the pSS subgroups compared to HC. The non-classical monocytes showed a weak decreasing pattern in the pSS patient subgroups.

### SSA- and SSA+ pSS patients show altered expression of HLA-DR and CD38 compared to healthy controls

**Figure 2:**
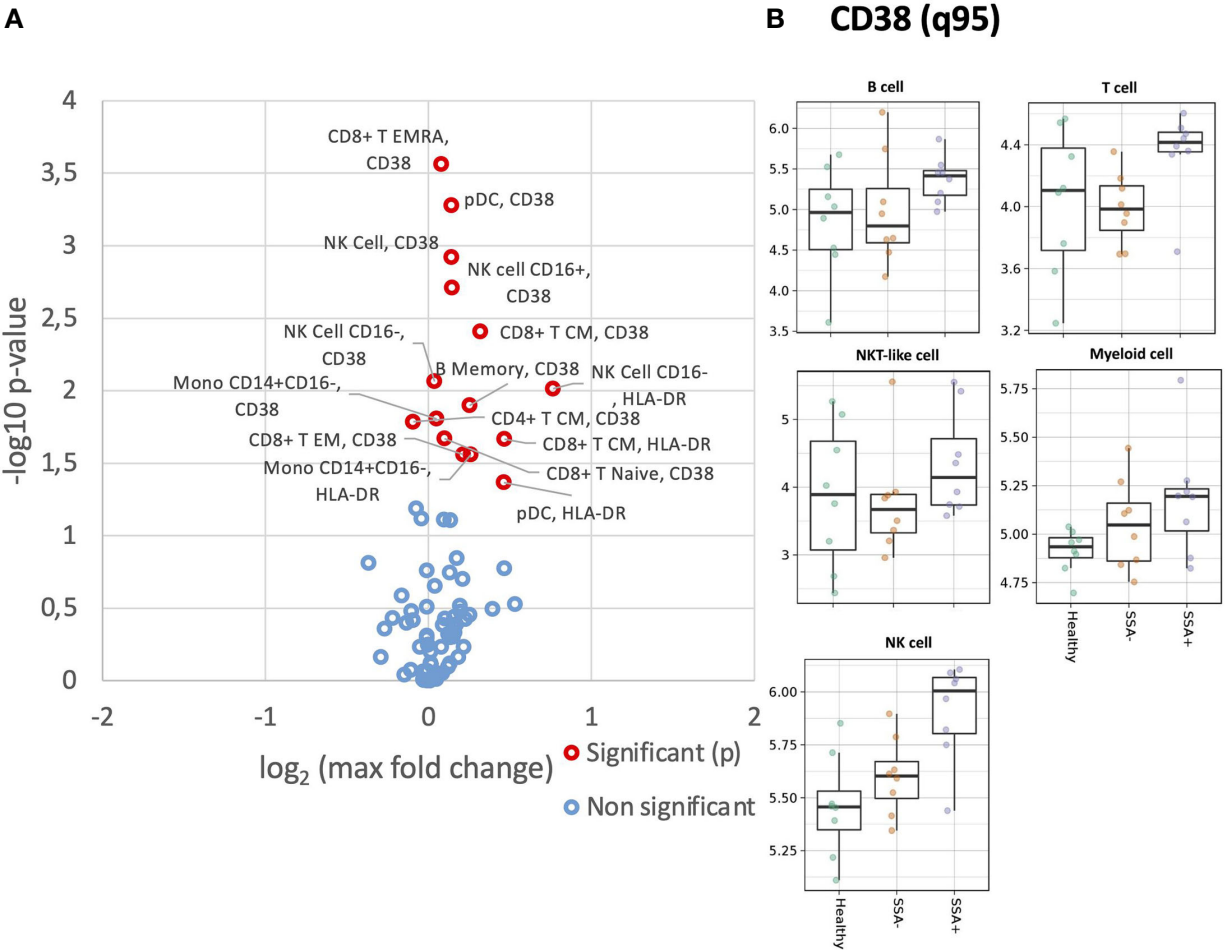
Altered expression of HLA-DR and CD38 on unstimulated CD45+ PBMC subsets in SSA- an SSA+ pSS patients compared to healthy donors. Assessment of the expression levels on different cell subsets of unstimulated PBMC was done by mass cytometry. Differential expression analysis was done using the limma R package. Comparisons were made among healthy controls (n=8), SSA- pSS patients (n=8) and SSA+ pSS patients (n=8) using the 95th quantile (q95). **(A)**, Overview of differential expression of activation markers in different cell subsets, with altered expression levels between the groups highlighted in red (p and FDR ≤ 0.05), with closed symbols indicating differences which were still significant after correcting for multiple testing and max fold change between groups given on the X-axis. **(B)**, Scatterplots highlighting widespread dysregulated expression of CD38. Medians are indicated for each box.

We next analyzed expression levels of HLA-DR and CD38 on the different cell populations. Compared to healthy donors, a slightly increased HLA-DR expression was detected in all major cell populations for the pSS patients, without much difference between the SSA- and SSA+ subgroups (**Supplementary Figure S1**). Analysis of HLA-DR expression in the various cell subpopulations revealed increased HLA-DR levels in memory B cells of both patient subgroups compared to HC, while the CD8+ T CM and EM subsets, CD4+ T EM cells, CD56+CD16+ NK cells, classical and non-classical monocytes showed an increasing tendency across the three groups (**Figure 2A**). Interestingly, in CD56+CD16- NK cells and pDCs, HLA-DR levels were elevated in both SSA- and SSA+ pSS patients compared to HC, with the highest expression in the SSA- patients (**Figure 2B**). None of the differences reached statistical significance.

B cells, T cells and NKT-like cells showed increased expression of CD38 in the SSA+ subgroup, while a slight increase across the three groups was observed for NK and myeloid cells (**Supplementary Figure S2**). Among the subsets, the increasing tendency was found in naïve B cells, CD56+CD16+ and CD56+CD16- NK cells and pDCs (**Figure 2B**). In memory B cells, cDCs and classical monocytes, CD38 expression was increased in SSA+ patients compared to HC and SSA- patients (**Figure 2B**). Increased levels of CD38 were also observed in the SSA+ patients in the majority of the CD8+ and CD4+ T cells subsets analyzed (**Supplementary Figure S3**).

### Elevated basal ERK1/2 levels in pSS patient subgroups compared to healthy donors

**Figure 3:**
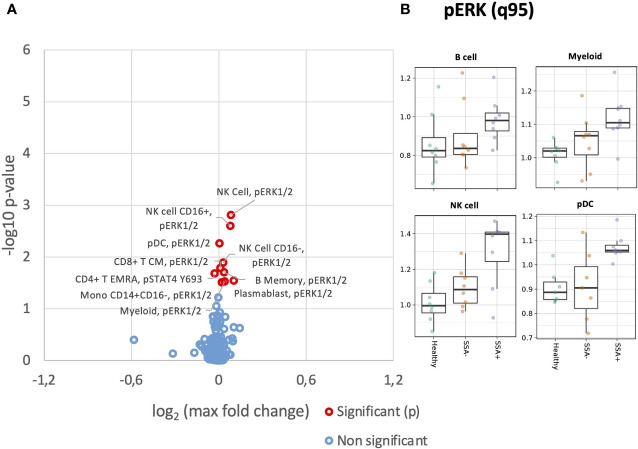
Altered basal expression of phospho-epitopes in unstimulated CD45+ PBMC subsets in SSA- an SSA+ pSS patients compared to healthy donors. Assessment of the phosphorylation levels on different cell subsets of unstimulated PBMC was done by mass cytometry. Differential expression analysis was done using the limma R package. Comparisons were made among healthy controls (n=8), SSA- pSS patients (n=8) and SSA+ pSS patients (n=8) using the 95th quantile (q95). **(A)**, Overview of differential expression of phosphorylation levels in different cell subsets, with altered expression levels between the groups highlighted in red (p and FDR ≤ 0.05), with closed symbols indicating differences which were still significant after correcting for multiple testing and max fold change between groups given on the X-axis. **(B)**, Scatterplots highlighting basal pERK levels, medians are indicated for each box.

We next analyzed basal phosphorylation levels of MAPK/ERK and JAK/STAT pathways. Increased basal phosphorylation levels of ERK1/2 were observed in numerous cell subsets, even if not statistically significant after correction for multiple testing (**Figure 3A**). This increase appeared non-specific, with uncorrected significant increases of basal phosphorylation observed in myeloid, B cell, NK cells and T cell subsets. These increases however were mainly associated with SSA+ patients (**Figure 3B**).

### Aberrations in phospho-signaling in the immune cells of SSA- and SSA+ pSS patients upon IFNα2b stimulation

**Figure 4:**
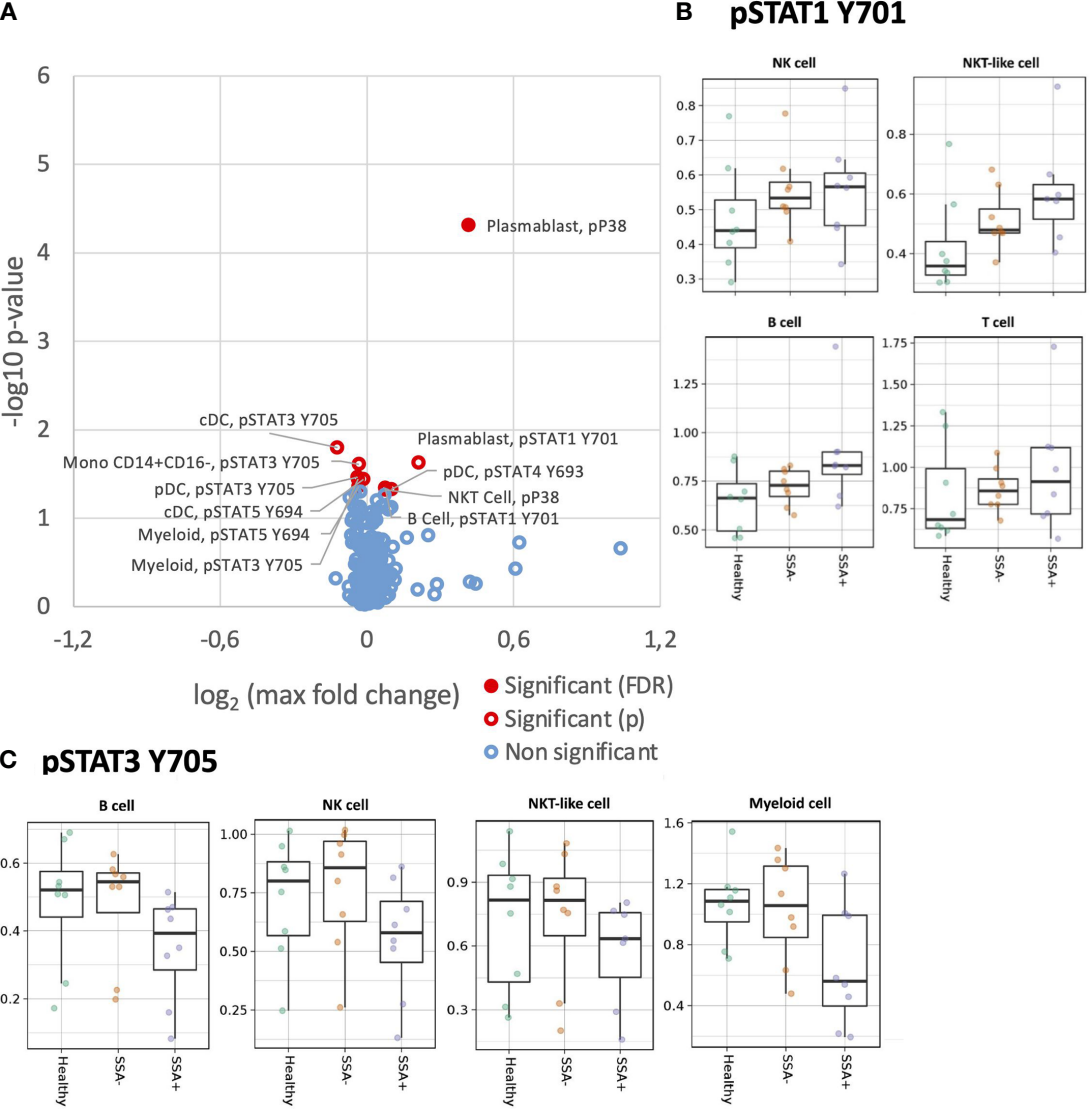
Altered IFNα2b induction of intracellular signalling in CD45+ PBMC subsets in SSA- an SSA+ pSS patients compared to healthy donors. Assessment of the phosphorylation levels on different cell subsets of IFNα2b stimulated PBMC was done by mass cytometry. Differential expression analysis was done using the limma R package. Comparisons were made among healthy controls (n=8), SSA- pSS patients (n=8) and SSA+ pSS patients (n=8) using fold changes in expression levels between unstimulated and the corresponding stimulated sample. Fold change was calculated as the log base 2 of the q95 dual count measurement for the stimulated value of interest (cell subtype, phosphor-epitope) minus – the log base 2 of the q95 for the corresponding unstimulated sample. **(A)**. Overview of differential cell signalling activation markers in different cell subsets, with altered fold change between the groups highlighted in red (p and FDR ≤ 0.05), with closed symbols indicating differences which were still significant after correcting for multiple testing and max fold change between groups given on the X-axis. **(B, C)** Scatterplots highlighting dysregulated IFNα2b induced phosphorylation of STAT1Y701 and STAT3Y705, medians are indicated for each box.

As we previously found alterations in the MAPK/ERK and JAK/STAT pathways in T, B and NK cells of pSS patients upon stimulation with IFNα2b (25), we wanted to confirm these results and further define the cellular subpopulations responsible for the differences. Significant opposing responses to IFNα2b were observed in diverse cellular subtypes for tyrosine residues (**Figure 4A**), with decreased STAT3 Y705 and STAT5 Y694, and increased STAT1 Y701 responses notable.

Although not reaching significance, a general increased response was also observed for pSTAT1 Y701 in B, T, NK and NKT-like cell subsets in the pSS patients, with maximum fold change seen in the SSA+ subgroup (**Supplementary Figure S4**). On analyzing the different cellular subpopulations, memory and naïve B cells, CD56+CD16+ NK cells, cDCs and pDCs showed a increased response to pSTAT1 Y701 in the pSS patients with SSA+ patients having the highest fold-changes (**Figure 4**). Various T cell subsets, like the CD8+ naïve, CM and T EMRA cells, CD4+ naïve, CM and EM subsets, showed a similar pattern (**Figure S5**).

For STAT3 Y705, a slightly decreased phosphorylation response was observed in B, NK, NKT-like and myeloid cells, in the SSA+ pSS patients compared to HC and SSA- patients (**Figure S6**). Further analysis of the cellular subtypes showed that memory and naïve B cells, both NK cell subsets, pDCs and classical monocytes exhibited this decreased fold change limited to SSA+ pSS patients (**Figure 4C**). No obvious differences were observed for pSTAT3 S727.

pSTAT4 Y693 was reduced in NK cells of pSS patients upon IFNα2b stimulation, even though this did not reach statistical significance. Interestingly, this was also seen in T cells for the SSA+ patients (**Supplementary Figure S7**). A slight reduction in STAT5 Y694 phosphorylation was observed in the B, T and myeloid populations upon IFNα2b stimulation (**Supplementary Figure S8**). Various subsets of T cells also showed a similar pattern (**Supplementary Figure S9**).

### Increased STAT1 Y701 response upon IFNγ stimulation in pSS patients

**Figure 5:**
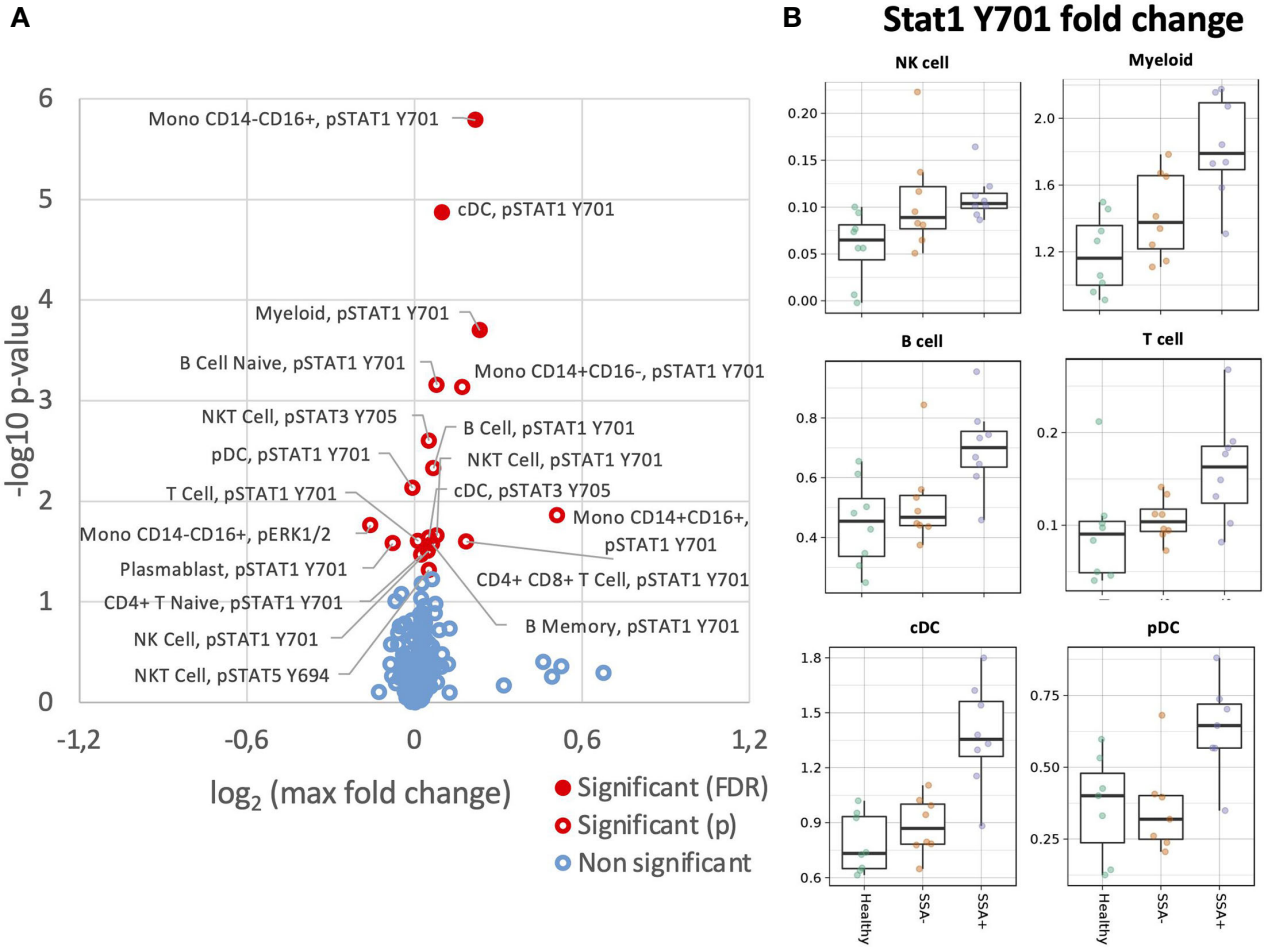
Altered IFNγ induction of intracellular signalling CD45+ PBMC subsets in SSA- an SSA+ pSS patients compared to healthy donors. Assessment of the phosphorylation levels on different cell subsets of IFNγ PBMC was done by mass cytometry. Differential expression analysis was done using the limma R package. Comparisons were made among healthy controls (n=8), SSA- pSS patients (n=8) and SSA+ pSS patients (n=8) using fold changes in expression levels between unstimulated and the corresponding stimulated sample. Fold change was calculated as the log base 2 of the q95 dual count measurement for the stimulated value of interest (cell subtype, phosphor-epitope) minus – the log base 2 of the q95 for the corresponding unstimulated sample. **(A)**, Overview of differential cell signalling activation markers in different cell subsets, with altered fold change between the groups highlighted in red (p and FDR ≤ 0.05), with closed symbols indicating differences which were still significant after correcting for multiple testing and max fold change between groups given on the X-axis. **(B)**, Scatterplots highlighting increased phospho STAT1 Y701 IFNγ responses, medians are indicated for each box.

We next analyzed MAPK/ERK and JAK/STAT signaling pathways in response to IFNγ stimulation (**Figure 5**). Unlike IFNα, the effect of IFNγ was restricted to mainly STAT1 Y701. Amongst the parent populations, increased phosphorylation of STAT1 Y701 was seen in the B cells and myeloid cells with the highest fold change in the SSA+ subgroup (**Supplementary Figure S10**). On analyzing the different subpopulations, a significantly increased fold change for pSTAT1 Y701 was observed on comparing HC and SSA+ pSS patients in cDCs (FDR = 0.014), classical and non-classical monocytes (FDR= 0.044 and 0.001 respectively, **Figure 5A**).

## Discussion

There are very few studies on the phospho-signaling profiles of pSS patients. In addition, not much is known about the unique aspects of IFNγ signaling in pSS pathogenesis. As mass cytometry uses metal-tagged antibodies instead of fluorophores, it circumvents the limitations of flow cytometry, namely spectral overlap and autofluorescence (27). Consequently, it can measure a large number of parameters simultaneously, at a single-cell level (28). Due to this advantage, we could investigate the different PBMC subpopulations and observe the changes in phospho-epitopes upon stimulation in many more cell types compared to previous investigations. To our knowledge, this is the first study where type I and type II signaling pathways were separately investigated in so many cell populations using mass cytometry.

We observed a general global shift in tyrosine kinase driven phosphorylation towards Stat1Y701 activation pathways, and away from STAT3, STAT4 and STAT5 pathways. We observed an increased trend for induction of STAT1 Y701 phosphorylation after stimulation with IFNα2b in several PBMC subsets, as previously reported by us and others in the parent populations (24, 25). Interestingly, a significant increase was also seen for IFNγ stimulation. Enhanced STAT1 Y701 signaling may be responsible for the IFN signature observed in some of the pSS patients. Cross-regulations between the STATs have also been observed, especially the downregulation of STAT1 by STAT3 and vice versa, *via* suppression of STAT1 homodimer formation and by competing for common docking sites (29, 30). Our analysis corroborated this reciprocal relationship, by revealing upregulated pSTAT1 Y701 induction and a reduced tendency for pSTAT3 Y705 induction by IFNα2b in most cell types. The same pattern was noted for pSTAT1 Y701 vs pSTAT5 Y694. STAT5 is involved in anti-apoptotic and mitogenic signaling (31). Our finding of a reduced tendency towards pSTAT5 Y694 induction again justifies the opposing nature of STAT functions. In contrast to our previous findings, there were no observable changes in STAT3 S727 phosphorylation levels upon stimulation (25). We also did not observe any significant differences in pSTAT6 Y694 induction.

While STAT4 contributes to IFNγ production and IFNγ responses, STAT1 negatively regulates it (32, 33). Increase in STAT1, during viral infections, by type 1 IFN or IFNγ, have been associated with decreased STAT4 access to receptors (34). Our analysis revealed a weak tendency for NK-cell specific reduction of pSTAT4 Y693 induction (both subsets) while increasing pSTAT1 Y701 (CD56+CD16+ subset), on IFNα2b stimulation. Such a profile may polarize these cells towards low IFNγ production and increased cell cytotoxicity (35). A similar pattern was also observed in the memory T cell subtypes, which might render them susceptible to decreased IFNγ production (36). Moreover, impaired CD8+ T cell expansion in response to viral infections has been reported earlier in STAT4-deficient mouse models, that is also associated with higher STAT1 levels (37). Our finding of reduced STAT4 vs increased STAT1 levels in the T cells may point towards proliferative and functional impairments.

We also found slightly increased basal phosphorylation levels of p38 in the different T cell subsets, CD56+CD16+ NK cells and non-classical monocytes in SSA+ patients. This confirms our previous findings of increased basal phosphorylated p38 in T and NK cells (25). p38 has been associated with TNFα, IFNγ and IL-1 production in T cells, monocytes and macrophages (38) and hence, elevated phosphorylation levels of p38 may explain the upregulations in some of these cytokines observed by us in pSS patients (39). However, as p38 can be activated by stress (40), our results should be interpreted with caution. In contrast to our previous finding, we saw increased basal phosphorylated ERK levels in most of the cell types analyzed. The important role of ERK in cell proliferation and differentiation is well known, with reduced ERK activity negatively affecting the activation of T cells (41). Previous studies in RA patients have revealed augmented ERK activity in hyperactive T cells (42). Defective ERK signaling has also been associated with SLE (43).

Differential leukocyte counts in pSS patients have been associated with higher titers of autoantibodies and disease severity (9, 11). Fluctuations in cell concentrations may arise due to migration from peripheral blood to the tissues or due to apoptosis (9, 44). Mariette et al. were the first to use mass cytometry for the analysis of pSS patients where they dissected the parent populations of PBMC into subsets and identified a disease-specific signature for pSS: decrease in CD4+ T cells, memory B cells and pDCs, with increase in activated CD4+ and CD8+ T cells and plasmablasts (9). They also found significantly reduced NK cells in the SSA+ patients. Using mass cytometry, we also identified the different cell subsets in PBMC and confirmed some of the previous findings, including decrease in memory B cells, CD56+CD16+NK cell subset, cDCs and pDCs as well as increase in classical monocytes (9, 11, 44, 45). However, contrary to our earlier findings, we observed a weak decreasing trend for non-classical monocytes and did not observe any significant reduction in NKT-like cells in the pSS patients (11). Further we found a slight increase in naïve B cells frequency in the patient subgroups, that was reported by us earlier (46). Although overall T cell lymphopenia was not observed, we detected an imbalance in the T cell subsets with a decrease in most of the T memory subpopulations in the SSA- and SSA+ patients, except CD8+ T EM and EMRA subsets. Activated T cells were identified for some of the memory populations, but we did not find any significant differences in their frequencies between the controls and patients. Plasmablasts and intermediate monocytes were identified, but their numbers were too low for any reliable statistical analysis. One major reason for some of the cell types not reaching significance is the low sample size of our experiment. Also, various studies have used different sampling methods, like whole blood versus PBMC and cryopreserved versus fresh, that may explain some of the discrepancies observed between our findings and previous reports. Another important aspect is the EGM and medication status of the patients. As our focus was on the impact of autoantibody on patient profiles, we enrolled only EGM negative and non-medicated patients, whereas in some of the other studies a portion of the patients were medicated and suffered from a variety of EGMs. EGMs have been associated with increased comorbidities and increased mortality in pSS patients compared to the general population (47, 48). Therefore, absence of EGM in our cohort may indicate a less severe form of the disease in our patients, contributing to differences with earlier observations.

Activated T cells contribute to pSS pathogenesis by producing proinflammatory cytokines and inducing B cell hyperactivation (49). Although differences in cell frequencies were undetectable, our analysis revealed an upregulated trend for the expression of activation markers, HLA-DR and CD38, in most of the CD8+ and CD4+ T memory cell subpopulations, particularly the SSA+ pSS patients. In general, we observed a more activated phenotype for many cell types analyzed, in the pSS patients compared to healthy donors, which was most noticeable in the SSA+ patients. CD38 is also important for leukocyte migration to inflamed tissues and optimum chemotactic responses of DCs (50). Therefore, increased CD38 may partly explain the observed leukocyte infiltration to the salivary glands from peripheral blood. Taken together, these might make the patients prone to enhanced secretion of pro-inflammatory cytokines (e.g., BAFF by pDCs and monocytes) and autoantibody production (51).

This study has a few limitations. First, our cohort size was small with few individuals (n=8) per group. This particularly affects high-dimensional datasets as corrections for multiple testing are needed for optimal statistical analysis of such data and a low sample size negatively affects the p value of individual parameters. So, it is probable that some of the cell types and phospho-proteins that showed a trend but did not reach statistical significance was due to the small size of our cohort. Due to a limitation in cell number acquired per individual, some cell populations with low frequencies could not be included in our analyses, even though particularly plasmablasts showed interesting trends in several of the phospho-proteins. Second, we excluded Tregs from our final analysis due to absence of FoxP3 in our panel, without which Tregs cannot be identified with confidence. In general, signals (quantile/q95) were quite low for many of the phospho-epitopes in our study. PBMC were stimulated for 12 minutes, which is not optimal for all the phospho-epitopes studied. Therefore, we chose to use 95^th^ quantile instead of median for our statistical analyses as it is more sensitive. In general, shifts in signals for the phospho-proteins are not large and the median, unlike the 95^th^ quantile, can miss minor changes that lie at the tail of the distribution. Also, we observed a clear batch effect, with low signals in batch 2 samples compared to the other three batches. It should be noted that the batch effect was not consistent and was more prominent for some phospho-markers in some cell types and less obvious or even absent in others. Hence, we decided not to perform any kind of normalization, and control for this by comparing within each batch. Fourth, the observed signaling patterns could be an effect of the shifts in cell frequencies, instead of true signaling differences. Finally, our study was based on cryopreserved PBMC. Although peripheral blood is a good indicator of the disease, the main site of disease activity, the salivary glands, should be investigated to get a comprehensive picture. Mariette and his group detected increased activated CD8+ T cells, fully differentiated plasma cells and activated glandular epithelial cells in pSS lip biopsy samples (9). Hence, examining the complex interactions between the infiltrating immune cells and the glandular epithelium, with the help of imaging mass cytometry, in pSS patients is of future interest.

## Conclusions

Enhanced activation status of certain cell types and increased response to type I and type II IFNs through pSTAT1 Y701, coupled with reduced pSTAT3 Y705 and pSTAT5 Y694 signaling, may predispose the pSS patients, particularly the SSA+ subgroup, to an increased expression of IFN-induced genes and autoantibody production. Hence, targeting these pathways may help in developing optimized treatment strategies for the patient subgroups.

## Materials and methods

Sample processing and analysis pipeline used in this study is shown in **Supplementary Figure S11**. All relevant information for repeating the experiment as presented in “The minimum information about a Flow Cytometry Experiment (MIFlowCyt) (52) is given in **Supplementary Table S2**.

### Study participants

16 female pSS patients were selected to be included in this study, based on the presence or absence of SSA autoantibodies (SSA-, n=8; SSA+, n=8), absence of EGM and no prescribed medication. All pSS patients fulfilled the American-European Consensus Group (AECG) diagnostic criteria and did not exhibit any additional autoimmune diseases or lymphoma (53). 8 female age-matched healthy controls (HC) were collected from the blood bank. An overview of the cohort characteristics is given in **Table 1**. All participants provided written informed consent and the study was approved by the regional ethical committee (#2009/686).

**Table 1:** Cohort characteristics *Positive focus score: ≥ 1 (≥ 50 cells per 4 mm^2^), ANA, anti-nuclear antibody; NA, not analysed.

	SSA - pSS patients	SSA + pSS patients	Healthy controls
**Cohort features:**			
Number	8	8	8
Age in years (Mean ± SD)	67.75 ± 12.2	57.63 ± 19.9	60.13 ± 11.3
Range	41-79	23-77	43-71
Female: Male	8:0	8:0	8:0
**Clinical features:**			
SSA+	0	8	
SSB+	0	4	
Both SSA+ and SSB+	0	4	
SSA+ and SSB-	0	4	
ANA+	1	8	
Extraglandular manifestations	0	0	
Focus score:			
Positive*	6	1	
Negative	0	1	
NA	2	6	
Medication	0	0	

### Blood sampling and PBMC isolation

Peripheral blood from pSS patients was collected in Lithium-heparin tubes (BD Diagnostics) at the Department of Rheumatology, Haukeland University Hospital, Bergen, Norway. Blood from age- and sex-matched healthy donors was collected at the blood bank at the Haukeland University Hospital in Bergen, Norway. PBMC were isolated by density gradient centrifugation using Lymphoprep™ (Axis-Shield, Oslo, Norway) and cryopreserved as described previously (54). For long-term storage, the cells were kept at -150°C.

### Sample preparation, stimulation and barcoding

Cells were thawed quickly in a water bath (37°C) and washed in serum-free medium, X-vivo-20™ (Lonza, Switzerland) containing Nuclease (1:10,000; Pierce™ Universal Nuclease for Cell Lysis, Thermo Fisher Scientific, MA, USA). Then, the cells were resuspended in X-vivo-20™ and allowed to rest in the incubator (37°C, 5% CO_2_) for 2 hours. Next, 2x10^6^ cells were transferred from each sample to three corresponding wells of a MegaBlock^®^ 96 deep well plate (Sarstedt, Germany). After centrifugation, cells were resuspended in RPMI-1640 without additives (Lonza, Switzerland) and incubated for 5 minutes (37°C) with live/dead marker Cell-ID™ Cisplatin (final concentration 2.5 μM). The cells were washed and resuspended in X-vivo-20™ and either left unstimulated or were stimulated with IFNα2b or IFNγ (final concentration 100ng/ml, ImmunoTools, Friesoythe, Germany) for 12 minutes in the incubator (37°C, 5% CO_2_). The cells were fixed with Maxpar Fix I Buffer for 10 minutes at RT and barcoded following Fluidigm’s protocol for Cell-ID™ 20-Plex Pd Barcoding Kit. Finally, all the 20 barcoded samples from the plate were combined in a 5 ml polystyrene round-bottom tube (BD Biosciences, MA, USA) for staining. As internal control (IC), one sample from the same donor was included in each batch to identify and correct for potential inter-assay variation. In that case, IFNα2b and IFNγ were used together for stimulation.

### Staining

9 x10^6^ barcoded cells were first stained in cell staining buffer (CSB) containing titrated amounts of antibodies against extracellular epitopes (**Table 2**) for 30 minutes at RT, the cells were then washed with CSB and cells permeabilized using chilled Methanol (-20°C, Merck, Germany) for 15 minutes on ice. For intracellular staining, the cells were washed and resuspended in CSB containing titrated amounts of antibodies (**Table 2**), for 30 minutes at RT. The cells were washed in CSB and fixed in 1.6% formaldehyde for 10 minutes at RT (16% Formaldehyde, Methanol-free, Pierce™, diluted with Maxpar Phosphate Buffered Saline/PBS). Finally, cells were washed and incubated overnight at 4°C in Cell-ID™ Intercalator–Ir (diluted in Maxpar Fix and Perm Buffer, final concentration 66.7 nM). The next day, the cells were washed twice in CSB and frozen in 90% fetal bovine serum (FBS) with 10% DMSO at -70°C. Sample preparation and staining of all samples were completed in four consecutive days using one barcode batch per day. After four weeks, each frozen sample was thawed on ice, washed with CSB containing nuclease followed by another wash in Maxpar Cell Acquisition Solution (CAS). Cells were left pelleted until acquisition. Unless otherwise mentioned, all products were from Fluidigm (California, USA).

**Table 2:** List of monoclonal antibodies used for mass cytometry.

Marker	Clone	Metal tag	Manufacturer	Staining step
**CD3**	UCHT1	170Er	Fluidigm	Extracellular
**CD4**	RPA-T4	145Nd	Fluidigm	Extracellular
**CD8**	RPA-T8	146Nd	Fluidigm	Extracellular
**CD11c**	Bu15	159Tb	Fluidigm	Extracellular
**CD14**	M5E2	160Gd	Fluidigm	Extracellular
**CD16**	3G8	209Bi	Fluidigm	Extracellular
**CD19**	HIB19	142Nd	Fluidigm	Extracellular
**CD20**	2H7	147Sm	Fluidigm	Extracellular
**CD25**	2A3	169Tm	Fluidigm	Extracellular
**CD27**	L128	167Er	Fluidigm	Extracellular
**CD38**	HIT2	172Yb	Fluidigm	Extracellular
**CD45**	HI30	154Sm	Fluidigm	Extracellular
**CD45RO**	UCHL1	165Ho	Fluidigm	Extracellular
**CD56**	NCAM16.2	149Sm	Fluidigm	Extracellular
**CD123**	6H6	151Eu	Fluidigm	Extracellular
**CD127**	A019D5	168Er	Fluidigm	Extracellular
**CD235a/b**	HIR2	141Pr	Fluidigm	Extracellular
**HLA-DR**	L243	174Yb	Fluidigm	Extracellular
**pP38 MAPK** **(T180/Y182)**	D3F9	156Gd	Fluidigm	Intracellular
**pERK1/2 (T202/Y204)**	D13.14.4E	171Yb	Fluidigm	Intracellular
**pNF-κB**	K10-895.12.50	166Er	Fluidigm	Intracellular
**pSTAT1 Y701**	58D6	153Eu	Fluidigm	Intracellular
**pSTAT3 S727**	49/P-STAT3	176Yb*	BD Pharmingen™	Intracellular
**pSTAT3 Y705**	4/p-Stat3	158Gd	Fluidigm	Intracellular
**pSTAT4 Y693**	38/p-Stat4	161Dy*	BD Pharmingen™	Intracellular
**pSTAT5 Y694**	47	150Nd	Fluidigm	Intracellular
**pSTAT6 Y641**	18/P-Stat6	175Lu	Fluidigm	Intracellular

*Antibodies conjugated inhouse with Maxpar^®^ X8 Antibody Labeling Kit.

### Cell acquisition and normalization

Prior to acquisition, cells were resuspended in CAS solution with a 1:10 concentration of EQ™ Four Element Calibration Beads. Samples were then passed through a 35µm cell-strainer (Falcon^®^, New York, USA) and acquired on a Helios™ Mass Cytometer with WB injector (Fluidigm Corporation, California, USA) at a speed of 300-500 events per second, at the Bergen Flow Cytometry Core Facility, University of Bergen, Norway. We collected at least 4x10^6^ events per barcode that translated to minimum 200,000 events per sample on average. The resulting FCS files were normalized to EQ beads (140Ce, 151Eu, 153Eu, 165Ho, 175Lu) and concatenated per barcode using the bead-based normalization algorithm in the Fluidigm CyTOF software.

### Data analysis

Normalized FCS files were uploaded to the Astrolabe Cytometry Platform (Astrolabe Diagnostics, Inc.) where transformation, debarcoding, data cleanup, unsupervised clustering, cluster labelling, visualization, cellular abundance, and differential expression analysis was done. Volcano plots of summary data (fold changes, p-values and FDR) were generated through Microsoft excel. Data cleanup (normalization bead and dead cell and doublet exclusion) and automatic debarcoding based on Pd staining combinations was conducted yielding 80 individual samples corresponding to each donor and stimulation combination. Cell subsets were identified in an approach known as “cell subset profiling”. In short, the cells within each sample were clustered based on similarity using the FlowSOM algorithm (55). The clusters were then identified as different known cell subsets using the Ek’Balam algorithm (56), a hierarchical-based algorithm that assigns cell subset labels through prior knowledge based gating, with subsets defined as per Maecker et al. (57), and Finak et al. (58). CD45+ populations were used as the starting population for clustering, CD45- being marked as debris. The cell populations identified are listed in **Table 3** and a detailed labeling hierarchy of all identified cell types is illustrated in **Supplementary Figure S12**. Heatmap of the expression of the surface markers in all the cell subsets from one sample is shown in **Supplementary Figure S13**. Differential abundance analysis of the cell subsets was done using the *edgeR* R package (59), with frequencies of cell subsets relative to the number of CD45+ cells in a sample used in calculations. In short, differential abundance in each cluster was evaluated using a negative binomial generalized linear model (NB-GLM) from the package to calculate log2 fold changes in cluster abundances between groups and associated multiple testing corrected FDR and uncorrected p-values. Pairwise comparisons between pairs (HC vs pSS patients, HC vs SSA- patients, HC vs SSA+ patients, SSA- vs SSA+ patients) and among the three groups (HC vs SSA- vs SSA+) were done. However, due to the low sample size we initially focused on the groupwise comparisons, with further comparison between subgroups made in parameters found to be significantly different between the groups (both unadjusted and adjusted (FDR) ≤ 0.05). Furthermore, the comparisons were done at two levels- the parent populations, i.e., the T and B lymphocytes, NK, NKT-like and myeloid cells as well as the subsets of these populations.

**Table 3:** Identified PBMC cell subsets and marker details.

Population name	CD markers
**B cells**	CD3-CD14- CD19+CD56-
B naive	CD27-
B memory	CD20+CD27+
Plasmablast	CD20-CD27+CD38+
**T cells**	CD3+CD14-CD19-CD56-
**Double negative**	CD4-CD8-
**CD4+**	CD4+CD8a-
Naive	CD27+CD45RO-
Central memory (CM)	CD27+CD45RO+
Effector memory (EM)	CD27-CD45RO+
Terminally differentiated (EMRA)	CD27-CD45RO-
**CD8+**	CD4-CD8a+
Naive	CD27+CD45RO-
Central memory (CM)	CD27+CD45RO+
Effector memory (EM)	CD27-CD45RO+
Terminally differentiated (EMRA)	CD27-CD45RO-
**NK cells**	CD3-CD14-CD19-CD56+
Natural killer (CD56+CD16+) (NK)	CD16+
Natural killer (CD56+CD16-) (NK)	CD16-CD56+
**NKT-like cells**	CD3+CD14-CD19-CD56+
**Dendritic cells**	CD3-CD14-CD19-CD56-
Conventional dendritic cells (cDC)	CD11c+CD16-CD123- HLA-DR+
plasmacytoid dendritic cells (pDC)	CD11c-CD123+HLA-DR+
**Monocytes**	CD3-CD19-
Classical monocytes	CD14+CD16-
Intermediate monocytes	CD14+CD16+
Non-classical monocytes	CD14-CD16+CD56-CD123-
	

Differential expression analysis was done using the *limma* R package (60) following the method for analysis of cytometric data as outlined in Weber et al. (61) For measurements of phosphorylation induction (pP38 MAPK, pERK1/2, pNF-κB, pSTAT1 Y701, pSTAT3 S727, pSTAT3 Y705, pSTAT4 Y693, pSTAT5 Y694 and pSTAT6 Y641) following either IFNγ or IFNα stimulations, fold changes were calculated as the log base 2 of the q95 dual count measurement for the stimulated value of interest (cell subtype, phosphor-epitope) minus the log base 2 of the q95 for the corresponding unstimulated sample. Expression of markers associated with cell activation (CD38, CD25 and HLA-DR) and basal phosphorylation were assessed using log base 2 of the q95 of the dual count value. The max log_2_ fold changes of medians between the 3 groups were further calculated to summarize differences between the groups.

## Data availability statement

The data that support the findings of this study are available on request from the corresponding author. The data are not publicly available due to privacy or ethical restrictions.

## Ethics statement

The studies involving human participants were reviewed and approved by Regional Health Authority West (#2009/686). The patients/participants provided their written informed consent to participate in this study.

## Author contributions

SA and RD conceptualized and designed the study. RD, SA and IS designed the mass cytometry panel. IS, SM, AA and AP processed the PBMC samples, IS and BB conducted the mass cytometry experiments. IS, AA, AJ, RD and Astrolabe Diagnostics processed and analyzed the data. RD, BB, JB and DH collected patient samples. IS and RD selected patients. IS, RD and SA drafted the manuscript. All authors revised the manuscript and approved the final version.

## Funding

This project was supported by the Broegelmann Foundation, the Western Norway Regional Health Authorities (grant nr. 912065) and the Meltzer Foundation. The funders did not play a role in the design of the study, collection, analysis, and interpretation of data and in writing the manuscript.

## Acknowledgments

We are thankful to all the patients and blood donors who participated in this study. We are grateful to Marianne Eidsheim and Kjerstin Jakobsen for their excellent technical support, and the staff at the laboratory at the Rheumatology clinics for the collection of blood samples from patients. The mass cytometry analysis was performed at the Flow Cytometry Core Facility, Department of Clinical Science, University of Bergen.

## Conflict of interest

The authors declare that the research was conducted in the absence of any commercial or financial relationships that could be construed as a potential conflict of interest.

## Publisher’s note

All claims expressed in this article are solely those of the authors and do not necessarily represent those of their affiliated organizations, or those of the publisher, the editors and the reviewers. Any product that may be evaluated in this article, or claim that may be made by its manufacturer, is not guaranteed or endorsed by the publisher.

## Abbreviations

cDCs, Conventional dendritic cells; CM, Central memory; EGM, Extraglandular manifestations; EM, Effector memory; FDR, False discovery rate; HC, Healthy controls; IFNs, Interferons; NK, Natural killer; PBMC, Peripheral blood mononuclear cells; pDCs, Plasmacytoid dendritic cells; pSS, Primary Sjögren’s syndrome; RT, Room temperature; SSA, Sjögren’s syndrome-related antigen A.
