# Supplementary material for: Aberrant signaling of immune cells in Sjögren’s syndrome patient subgroups upon interferon stimulation
Source: Front Immunol. 2022 Aug 22;13:854183. doi: 10.3389/fimmu.2022.854183 (PMC9441756; doi:10.3389/fimmu.2022.854183)
Supplement: Supplementary file 1 [file DataSheet_1.docx]

**Table S1:** Frequency of identified cell types in PBMC, determined by differential abundance analysis.

| Cell type | Frequency in heathy controls (%) | Frequency in SSA- pSS patients (%) | Frequency in SSA+ pSS patients (%) | FDR (groupwise comparison) |
| --- | --- | --- | --- | --- |
| Parent populations |  |  |  |  |
| B | 3.95 | 6.45 | 5.57 | 0.904959 |
| T | 74.23 | 74.1 | 73.12 | 0.904959 |
| NK | 13.67 | 10.05 | 9.19 | 0.238223 |
| NKT-like | 4.87 | 7.47 | 4.21 | 0.186436 |
| Myeloid | 7.17 | 8.0 | 7.38 | 0.238223 |
| Subsets |  |  |  |  |
| B memory | 1.37 | 1.04 | 0.55 | 0.017209 |
| B naive | 2.37 | 3.54 | 4.35 | 0.636605 |
| *Plasmablast | 0.07 | 0.06 | 0.09 | 0.963342 |
| CD4-CD8- T | 1.08 | 2.55 | 1.81 | 0.636605 |
| CD4+ T naive | 23.53 | 17.07 | 26.86 | 0.026949 |
| CD4+ T CM | 12.82 | 13.53 | 11.51 | 0.327483 |
| CD4+ T EM | 3.29 | 3.11 | 1.57 | 0.119383 |
| CD4+ T EMRA | 0.83 | 0.79 | 0.60 | 0.013158 |
| CD8+ T naive | 5.86 | 3.57 | 5.17 | 0.264916 |
| CD8+ T CM | 2.75 | 3.0 | 1.95 | 0.032339 |
| CD8+ T EM | 0.33 | 1.46 | 1.04 | 0.015962 |
| CD8+ T EMRA | 3.2 | 4.08 | 5.74 | 0.116686 |
| NK (CD56+CD16+) | 12.19 | 9.2 | 7.02 | 0.116686 |
| NK (CD56++CD16-) | 0.93 | 0.88 | 0.96 | 0.963342 |
| cDC | 1.01 | 0.66 | 0.55 | 0.861446 |
| pDC | 0.48 | 0.34 | 0.23 | 0.299438 |
| Classical monocyte | 3.14 | 5.8 | 5.69 | 0.062373 |
| *Intermediate monocyte | 0.04 | 0.12 | 0.09 | 0.978923 |
| Non-classical monocyte | 0.89 | 0.82 | 0.71 | 0.963342 |
|  |  |  |  |  |

* Cells not included in the final analyses

**Table S2.** Relevant information for repeating the experiment as presented in “The minimum information about a Flow Cytometry Experiment (MIFlowCyt)”.

| Data set | Sample/Reagent/  Controls/Instrument | Details |
| --- | --- | --- |
| Samples/specimens | Patient samples | *Species: Homo Sapiens, Sex: female, Mean age (range): 63 (23-79), Phenotype: pSS, Location: Department of Rheumatology, Haukeland University Hospital, Bergen, Norway. Collection methodology: Venule puncture* |
|  | Healthy donor samples | *Species: Homo Sapiens, Sex: female, Mean age (range): 60 (43-71), Phenotype: unknown, Location: Department of Rheumatology, Haukeland University Hospital, Bergen, Norway. Collection methodology: Venule puncture* |
|  | Single donor control | *Species: Homo Sapiens, Sex: unknown, Age: unknown, phenotype: unknown, Location:*  *Bergen, Norway. Collection methodology: Venule puncture* |
|  |  |  |
| Sample treatment | Blood collection | Lithium-heparin tubes for patients and healthy donor samples, and a citrate phosphate dextrose adenine blood collection bag for the single donor control |
|  | PBMC isolation | PBMC isolated within 1hr of blood collection by density gradient centrifugation with lymphoprep (Axis-Shield, Cat#07861) |
|  | Cryopreservation | *Immediately following isolation, PBMC samples were washed 2x with PBS (Lonza, Cat# 17-516F) and frozen at 5x10^6^ cells/ml in 7.5% DMSO (Sigma-Aldrich, Cat#D2650), 50% X-vivo 20^TM^ (Lonza, cat#BE04-448Q), 42.5% ProfreezeTM CDM NAO media (Lonza, cat# 12-769E) and at a cooling rate of 1 Celsius/min(CoolCell LX, Biocison, Cat#BCS-405) at -70°C overnight before long term storage in -150°C freezer* |
|  | Thawing | *PBMCs were thawed rapidly at 37°C in a water bath, 1ml of 37°C X-vivo 20^TM^ containing nuclease (1:10,000; Pierce^TM^ Universal Nuclease for Cell Lysis, Thermo Fisher Scientific, MA, USA) was added dropwise to each vial prior to addition to 9ml X-vivo 20^TM^ with nuclease and washed (300g) prior to culturing* |
|  | Culturing | *The PBMCs were cultured at 37°C with 5% CO2 at 3 x10 cell/ml for 2hrs in X-vivo 20^TM^ before being transferred to a Megablock® 96 well plate (Starstedt, Cat#82.1972.002).* |
|  | Live/ dead staining | *PBMCs were resuspended in RPMI-1640 (Lonza, Switzerland) and incubated for 5 minutes (37°C) with live/dead marker Cell-ID^TM^ Cisplatin (final concentration 2.5 μM, Fluidigm, SKU 201195)* |
|  | Stimulation | *The PBMCs were washed and resuspended in X-vivo-20^TM^ and either left unstimulated or were stimulated with IFNα2b or IFNγ (final concentration 100ng/ml, ImmunoTools, Friesoythe, Germany) for 12 minutes in the incubator (37°C, 5% CO2).* |
|  | Fixation | *Following stimulation PBMCs were immediately fixed at room temperature for 10 minutes with Maxpar Fix I Buffer (Fluidigm, SKU 201065) for 10 minutes at RT* |
|  | Transient permeabilization and Pd barcoding | *After fixation PBMCs were washed at 1000g for 5 min 4°C with barcode permeabilization buffer (Fluidigm, SKU 201057) twice and each sample (n=20) stained with a unique combination of 6 palladium isotopes from Cell-ID™ 20-Plex Pd Barcoding Kit (Fluidigm, SKU 201060) diluted in* *barcode permeabilization buffer and stained for 30 minutes at RT.* |
|  | SM Antibody staining | *Barcoded cells were then washed 2x in cell staining buffer (CSB, Fluidigm, SKU 201068) before being combined into a single sample. 9 x10^6^ barcoded cells stained in CSB containing titrated amounts of antibodies against extracellular epitopes for 30 minutes at room temperature.* |
|  | SM Antibody panel and staining concentrations | *Monoclonal antibodies- 170Er conjugated anti-CD3 (UCHT1, SKU 3170001B, dilution- 1:800), 145Nd conjugated anti-CD4 (RPA-T4, SKU 3145001B, dilution- 1:200), 146Nd conjugated anti-CD8 (RPA-T8, SKU 3146001B, dilution- 1:800), 159Tb conjugated anti-CD11c (Bu15, SKU 3159001B, dilution- 1:800), 160Gd conjugated anti-CD14 (M5E2, SKU 3160006B, dilution- 1:200), 209Bi conjugated anti-CD16 (3G8, SKU 3209002B, dilution- 1:200), 142Nd conjugated anti-CD19 (HIB19, SKU 3142001B, dilution- 1:400), 147Sm conjugated anti-CD20 (2H7, SKU 3147001B, dilution- 1:400), 169Tm conjugated anti-CD25 (2A3, SKU 3169003B, dilution- 1:200), 167Er conjugated anti-CD27 (L128, SKU 3167002B, dilution- 1:400), 172Yb conjugated anti-CD38 (HIT2, SKU3172007B, dilution- 1:200), 154Sm conjugated anti-CD45 (HI30, SKU 3154001B, dilution- 1:400), 165Ho conjugated anti-CD45RO (UCHL1, SKU* *3165011B, dilution- 1:400), 149Sm conjugated anti-CD56 (NCAM16.2, SKU* *3149021B, dilution- 1:1000), 151Eu conjugated anti-CD123 (6H6, SKU 3151001B, dilution- 1:400), 168Er conjugated anti-CD127 (A019D5, SKU 3168017B, dilution- 1:100), 141Pr conjugated anti-CD235a/b (HIR2, SKU 3141001B, dilution- 1:800), 174Yb conjugated anti-HLA-DR (L243, SKU 3174001B, dilution- 1:400). All from Fluidigm* |
|  | Permeabilization | *After extracellular staining the PBMC were washed in CSB, chilled (-20°C) methanol (Sigma Aldrich, cat#32213-2.5L-M) was added dropwise and the cells permeabilized for 15 minutes on ice.* |
|  |  | *Permeabilized PBMC were then washed in CSB and resuspended in CSB containing titrated amounts of antibodies against extracellular epitopes for 30 minutes at room temperature.* |
|  | IC Antibody panel and staining concentrations | *Monoclonal antibodies- 156Gd conjugated anti-pP38 MAPK (D3F9, SKU 3156002A, dilution- 1:150), 171Yb conjugated anti-pERK1/2 (D13.14.4E, SKU 3171010A, dilution- 1:100), 166Er conjugated anti-pNF-κB (K10-895.12.50, SKU 3166006A, dilution- 1:100), 153Eu conjugated anti-pSTAT1 Y701 (58D6, SKU 3153003A, dilution- 1:200), 158Gd conjugated anti-pSTAT3 Y705 (4/p-Stat3, SKU 3158005A, dilution- 1:200), 150Nd conjugated anti-pSTAT5 Y694 (47, SKU 3150005A, dilution- 1:200), 175Lu conjugated anti-pSTAT6 Y641 (18/P-Stat6, SKU 3175005A, dilution- 1:100). All from Fluidigm. 176Yb conjugated anti-pSTAT3 S727 (49/P-STAT3, dilution- 0.63* *µg/ml) and 161Dy conjugated anti-pSTAT4 Y693 (38/p-Stat4, SKU 3170001B, dilution- 5µg/ml), Antibodies custom made from BD Pharmingen^TM^ and antibody conjugation kits through Fluidigm (SKU 201176A and 201161A respectively).* |
|  | Fixation and Intercalator-Ir staining | *PBMC were then washed in CSB, fixed with 1.6% formaldehyde (16% Formaldehyde, Methanol-free, Pierce™, Cat#15710, diluted with Maxpar Phosphate Buffered Saline, Fluidigm, SKU 201058) for 10 minutes, washed in CSB and stained with Cell-ID™ Intercalator–Ir diluted in Maxpar Fix and Perm Buffer (Both Fluidigm, SKU 201192B and 201067 respectively) at a final concentration 66.7 nM overnight at 4°C.* |
|  | Freezing | *The following day PBMC were washed twice in CSB and frozen in 90% fetal bovine serum with 10% DMSO (Sigma, Cat#D2650) at -70°C until acquistion* |
|  | Acquisition | *Frozen samples were thawed on ice, washed with CSB containing nuclease, and washed in Maxpar Cell Acquisition Solution (CAS, Fluidigm, SKU 201240). PBMC were left pelleted until acquisition* |
|  |  |  |
| Controls | Mass cytometer tuning and calibration | *A standardized tuning and calibration procedure as outlined in the Helios user manual is followed daily and prior to sample acquisition. The mass cytometer is calibrated using tuning solution (Fluidigm, SKU 201072) to maximize the signal of Tm169 or Tb159 while keeping Gd155 (oxide; La139+O16) signal under 3% of the higher value. EQ Four Element Calibration Beads (Fluidigm, SKU 201078) are used to check sensitivity and signal stability.* |
|  | Signal normalization | *FCS files generated are normalized to EQ Four Element Calibration Beads using the bead-based normalization algorithm in the Fluidigm CyTOF software to control for signal variation.* |
|  | Single donor control | *Cryopreserved PBMCs from a single donor were included in each assay (n = 4) stimulated (IFNα and IFNγ) and unstimulated to assess assay variability.* |
|  | Biological control | *For each donor an unstimulated PBMC sample was analyzed to assess donor specific changes in the phosphorylation of phospho-epitopes following IFNα and IFNγ stimulation* |
|  |  |  |
| Instrument | Instrument type | *Helios™ (Fluidigm)* |
|  | Software | *CYTOF SOFTWARE V7.0 (Fluidigm)* |
|  |  |  |
| Data Analysis | Debarcoding, cell clustering and cell subset labelling | *All subsequent FCS file processing and data analysis was conducted through the* *Astrolabe Cytometry Platform (Astrolabe Diagnostics, Inc.). Each sample in a 20-plex barcode was identified based on its unique combination of palladium isotopes. Similar cells in a sample were then clustered into cell subsets using the FlowSOM algorithm and cell subsets were identified using the Ek'Balam algorithm.* |
|  | Abundance and expression analysis | *Differential abundance analysis of the cell subsets was done using the edgeR R package anddDifferential expression analysis was done using the limma R package* |
|  |  |  |

**
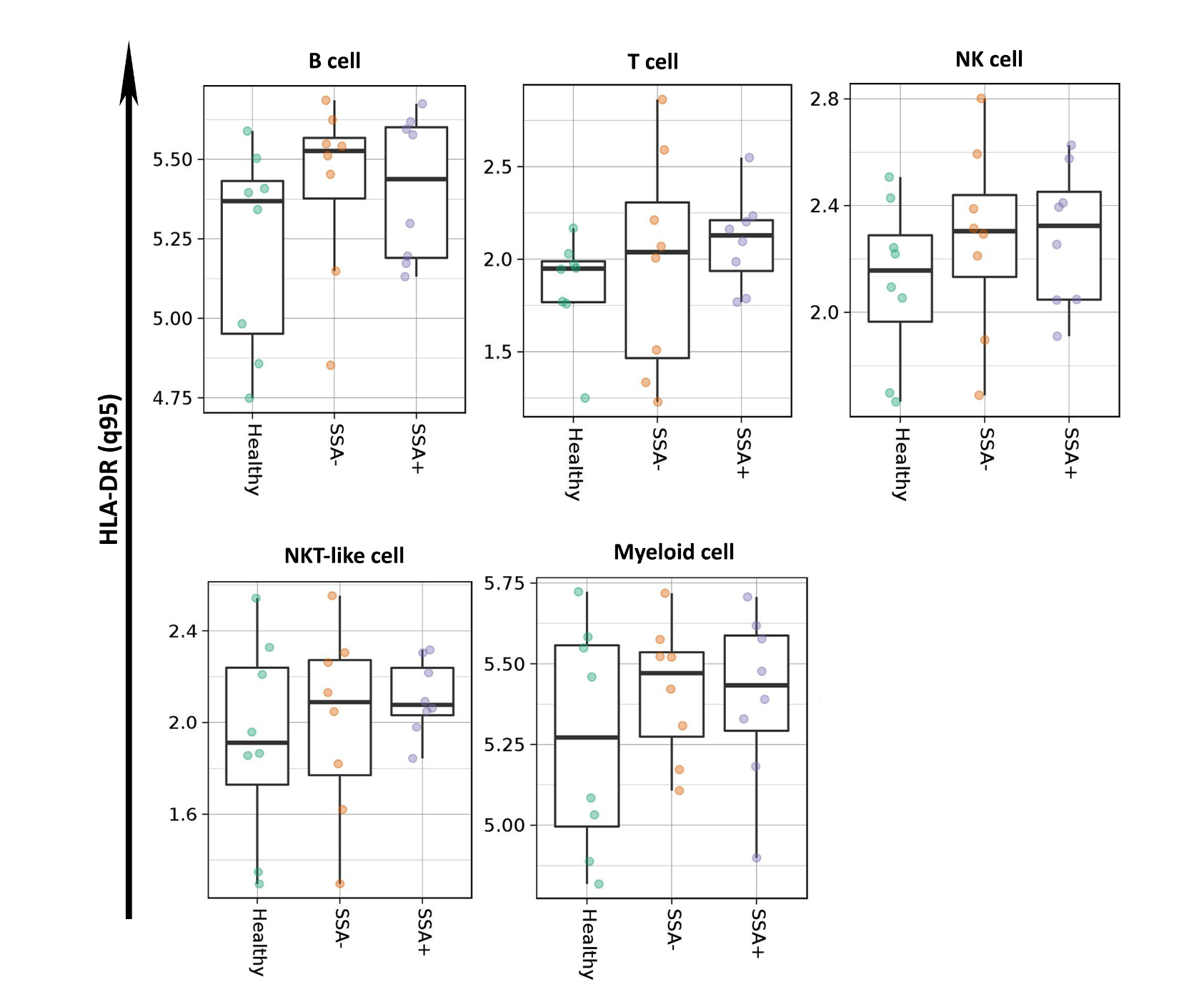
**

**Figure S1. Increased expression of HLA-DR in the parent populations of immune cells of SSA- and SSA+ pSS patients compared to healthy donors.** Assessment of the expression levels of HLA-DR on unstimulated PBMC parent populations was done by mass cytometry. Differential expression analysis was done using the limma R package. Comparisons were made among healthy controls/Healthy (n=8, green circles), SSA- pSS patients (n=8, orange circles) and SSA+ pSS patients (n=8, purple circles) using the 95^th^ quantile (q95). Medians are indicated for each box and values were considered statistically significant for FDR/adjusted p < 0.05.

**
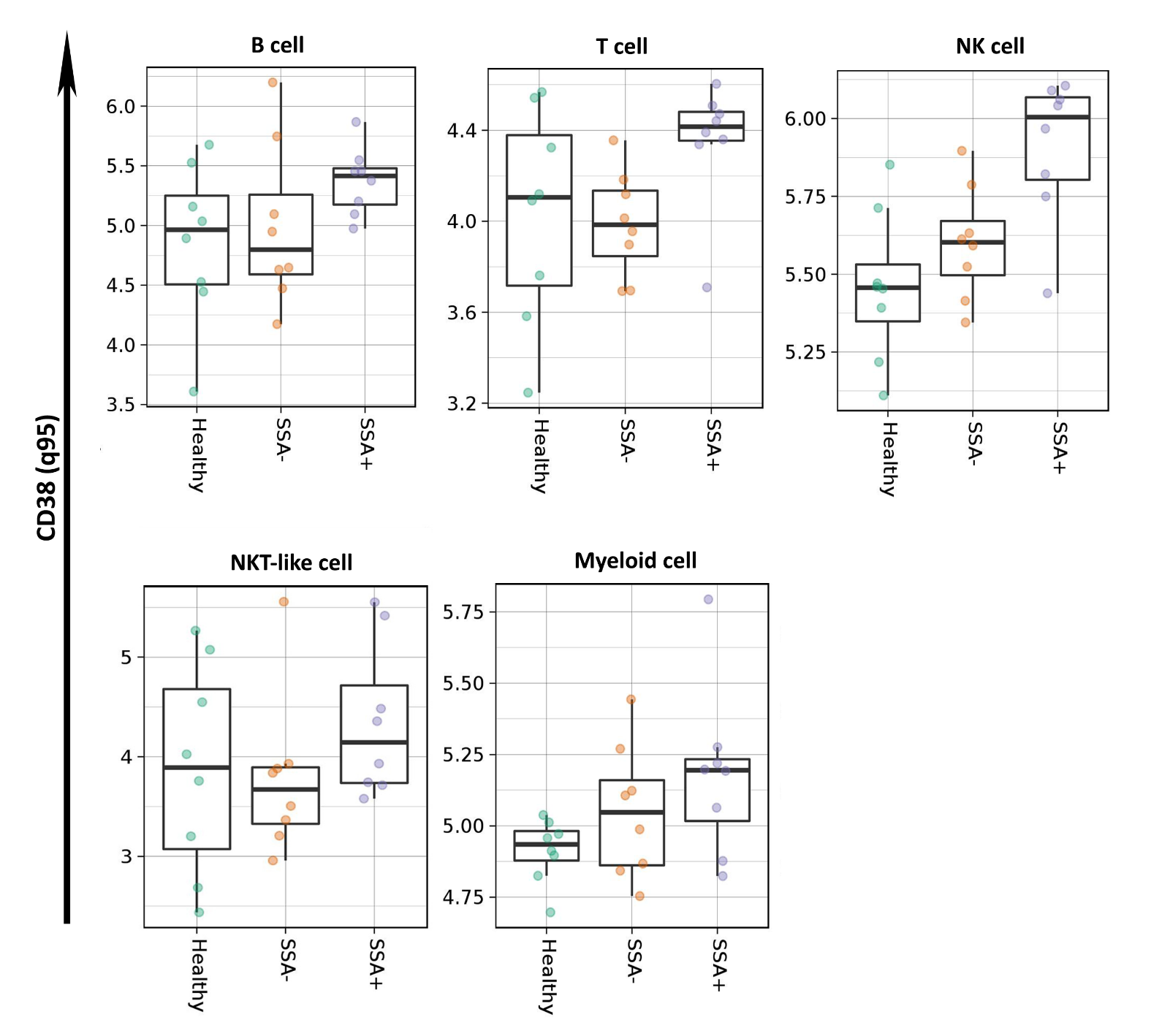
**

**Figure S2. Differential expression of CD38 in the parent populations of immune cells among healthy donors, SSA- and SSA+ pSS patients.** Assessment of the expression levels of CD38 on unstimulated PBMC parent populations was done by mass cytometry. Differential expression analysis was done using the limma R package. Comparisons were made among healthy controls/Healthy (n=8, green circles), SSA- pSS patients (n=8, orange circles) and SSA+ pSS patients (n=8, purple circles) using the 95^th^ quantile (q95). Medians are indicated for each box and values were considered statistically significant for FDR/adjusted p < 0.05.

**
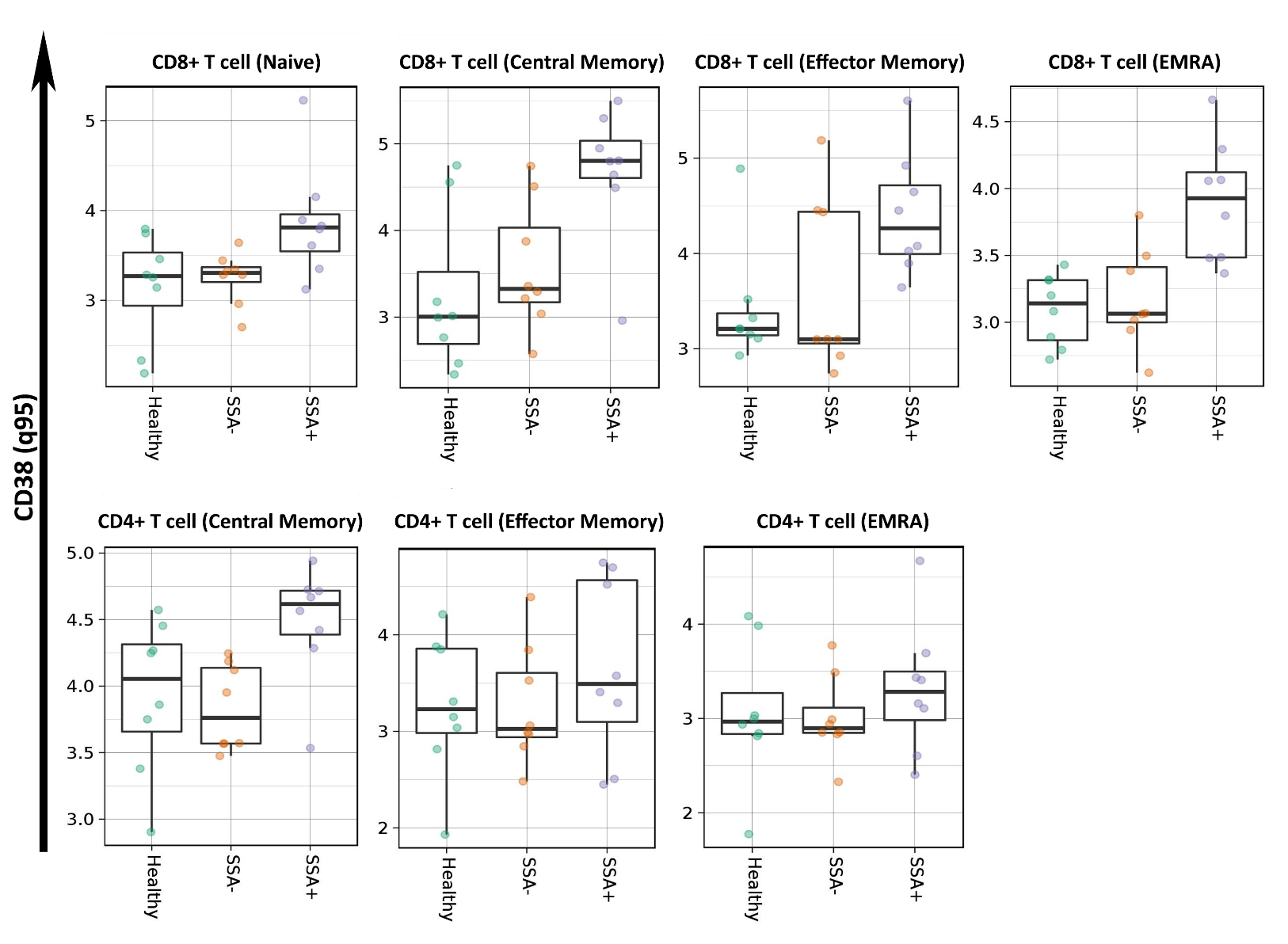
**

**Figure S3. Altered expression of CD38 in T cell subsets of SSA- and SSA+ pSS patients compared to healthy donors.** Assessment of the expression levels of CD38 on unstimulated T cell sub-populations was done by mass cytometry. Differential expression analysis was done using the limma R package. Comparisons were made among healthy controls/Healthy (n=8, green circles), SSA- pSS patients (n=8, orange circles) and SSA+ pSS patients (n=8, purple circles) using the 95^th^ quantile (q95). Medians are indicated for each box and values were considered statistically significant for FDR/adjusted p < 0.05.

**
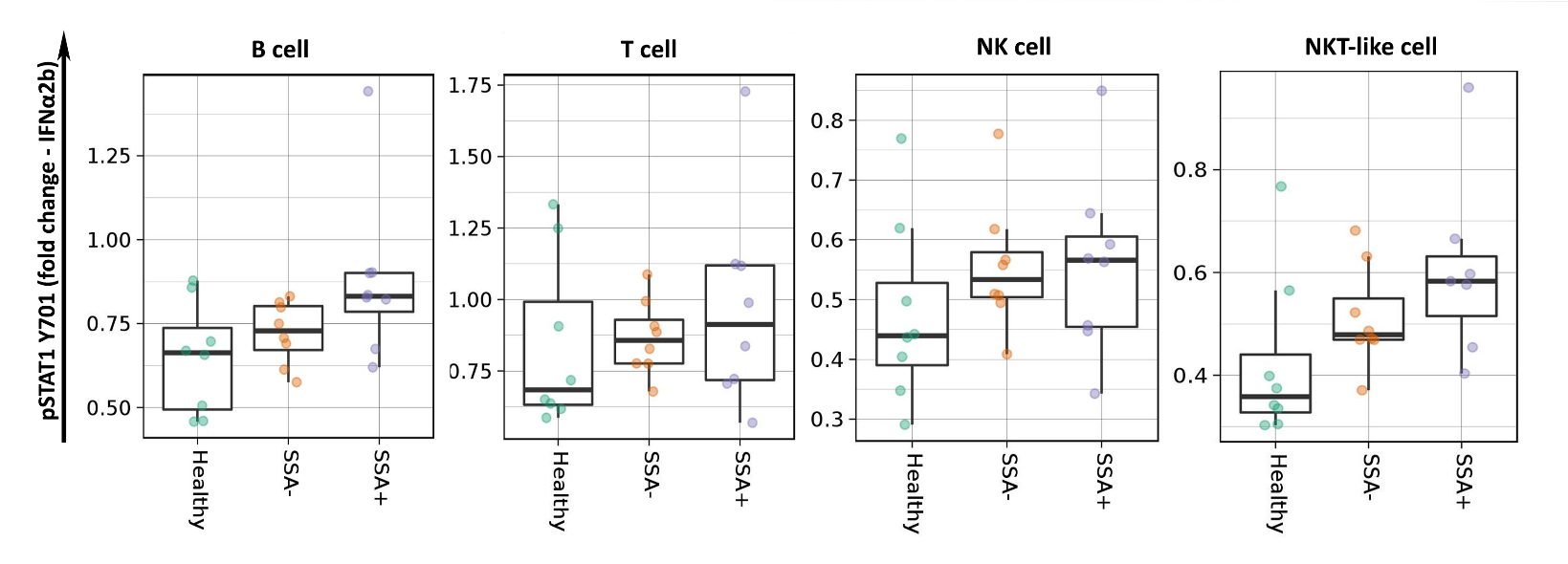
**

**Figure S4. Enhanced pSTAT1 Y701 signaling in the parent populations of PBMC in SSA- and SSA+ pSS patients compared to controls, upon IFNα2b stimulation.** Using mass cytometry, the level of pSTAT1 Y701 induction upon IFNα2b stimulation was evaluated in the parent populations of PBMC. Comparisons were made among healthy controls/Healthy (n=8, green circles), SSA- pSS patients (n=8, orange circles) and SSA+ pSS patients (n=8, purple circles). Differential expressions were analyzed using the limma R package and are expressed in terms of fold change. The median fold changes are indicated for each box and values were considered statistically significant for FDR/adjusted p < 0.05.

**
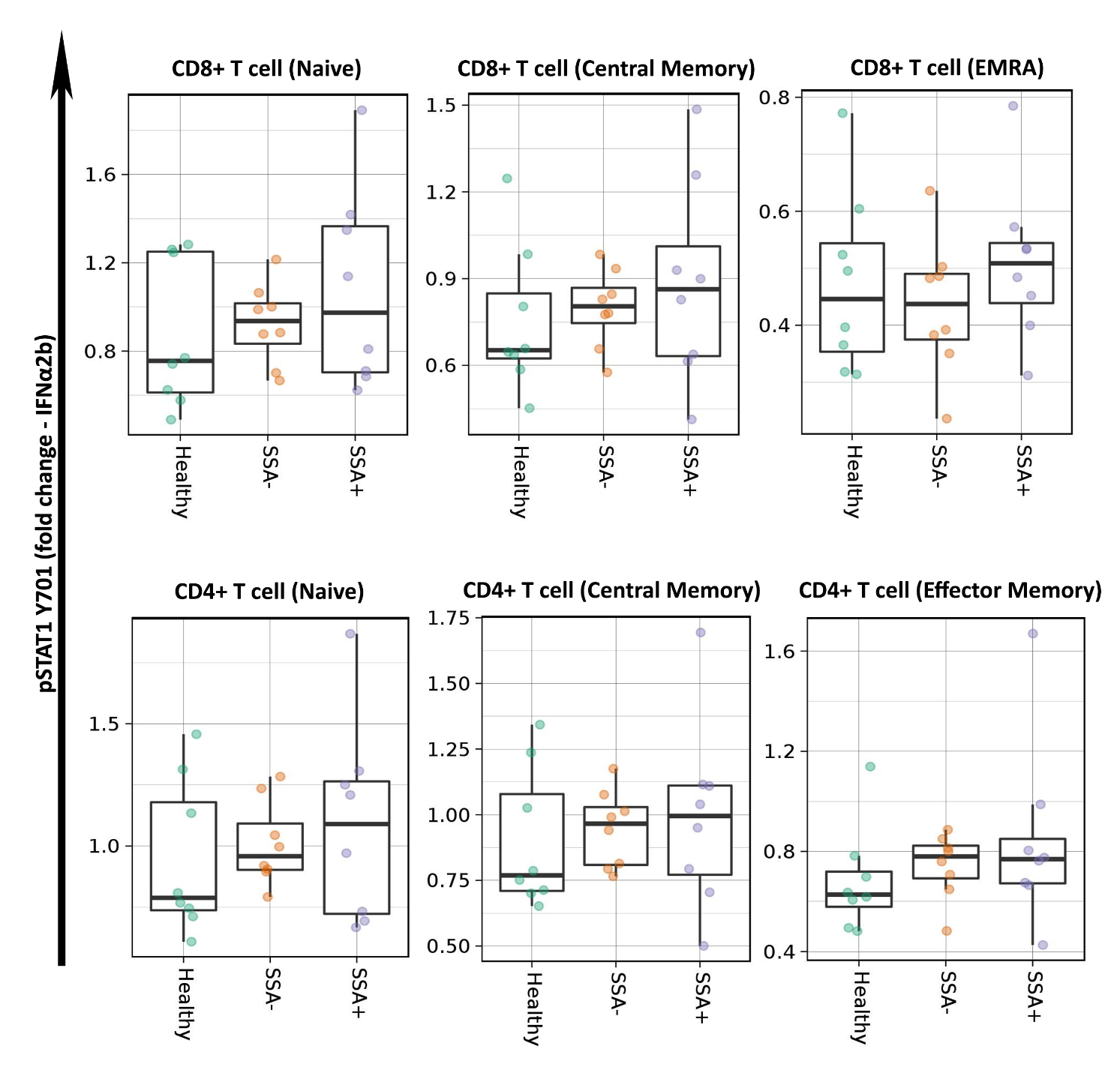
**

**Figure S5. Enhanced pSTAT1 Y701 signaling in the T cell subsets of SSA- and SSA+ pSS patients compared to controls, upon IFNα2b stimulation.** Using mass cytometry,the level of pSTAT1 Y701 induction by IFNα2b stimulation was evaluated in the different T cell subsets of PBMC. Comparisons were made among healthy controls/Healthy (n=8, green circles), SSA- pSS patients (n=8, orange circles) and SSA+ pSS patients (n=8, purple circles). Differential expressions were analyzed using the limma R package and are expressed in terms of fold change. The median fold changes are indicated for each box and values were considered statistically significant for FDR/adjusted p < 0.05.

**
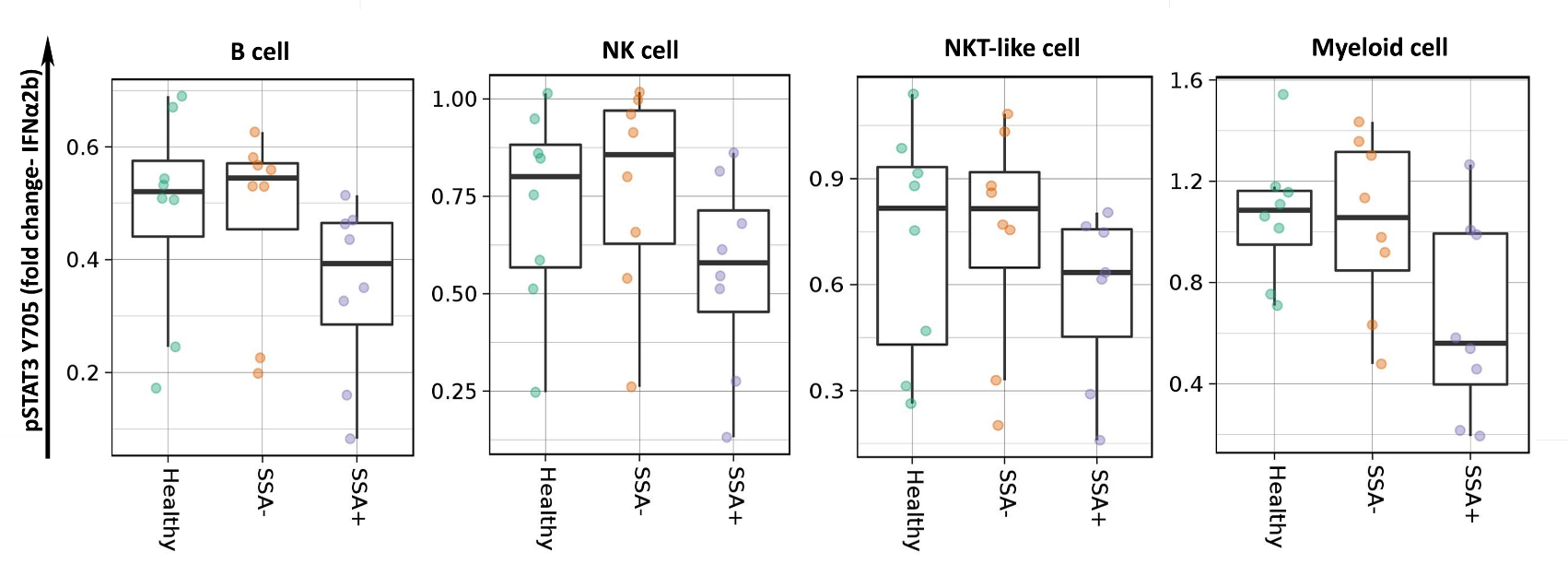
**

**Figure S6. Aberrations in phospho-signaling of STAT3 Y705 in the parent populations of PBMC in SSA- and SSA+ pSS patients compared to healthy donors, upon IFNα2b stimulation.** Levels of pSTAT3 Y705 induction upon IFNα2b stimulation were assessed by mass cytometry. Comparisons were made among healthy controls/Healthy (n=8, green circles), SSA- pSS patients (n=8, orange circles) and SSA+ pSS patients (n=8, purple circles). Differential expressions were analyzed using the limma R package and are expressed in terms of fold change. Median fold changes are indicated for each box and values were considered statistically significant for FDR/adjusted p < 0.05.

**
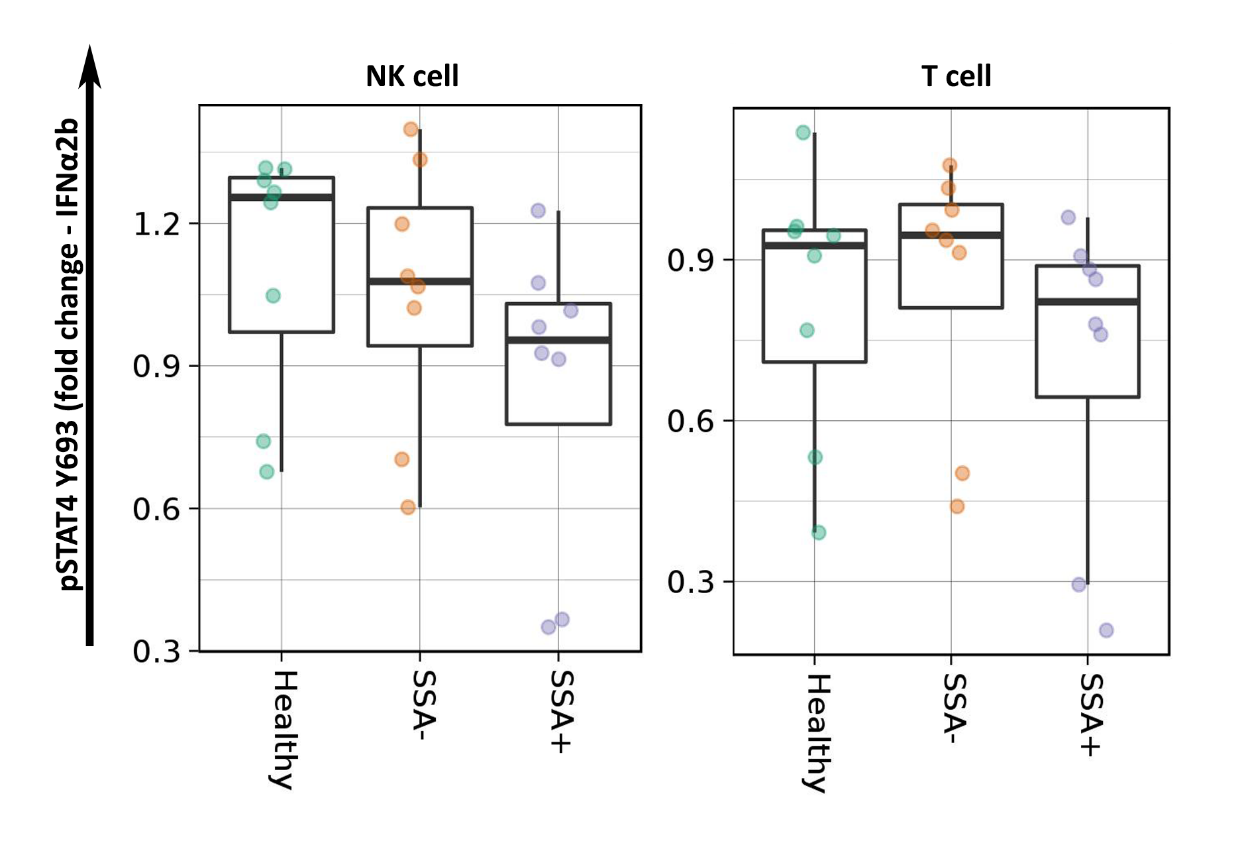
**

**Figure S7. Alterations in pSTAT4 Y693 signaling in the NK and T cells of SSA- and SSA+ pSS patients compared to healthy controls, upon IFNα2b stimulation.** Assessment of the level of pSTAT4 Y693 induction upon IFNα2b stimulation was done by mass cytometry. Comparisons were made among healthy controls/Healthy (n=8, green circles), SSA- pSS patients (n=8, orange circles) and SSA+ pSS patients (n=8, purple circles). Differential expressions were analyzed using the limma R package and are expressed in terms of fold change. Median fold changes are indicated for each box and values were considered statistically significant for FDR/adjusted p < 0.05.

**
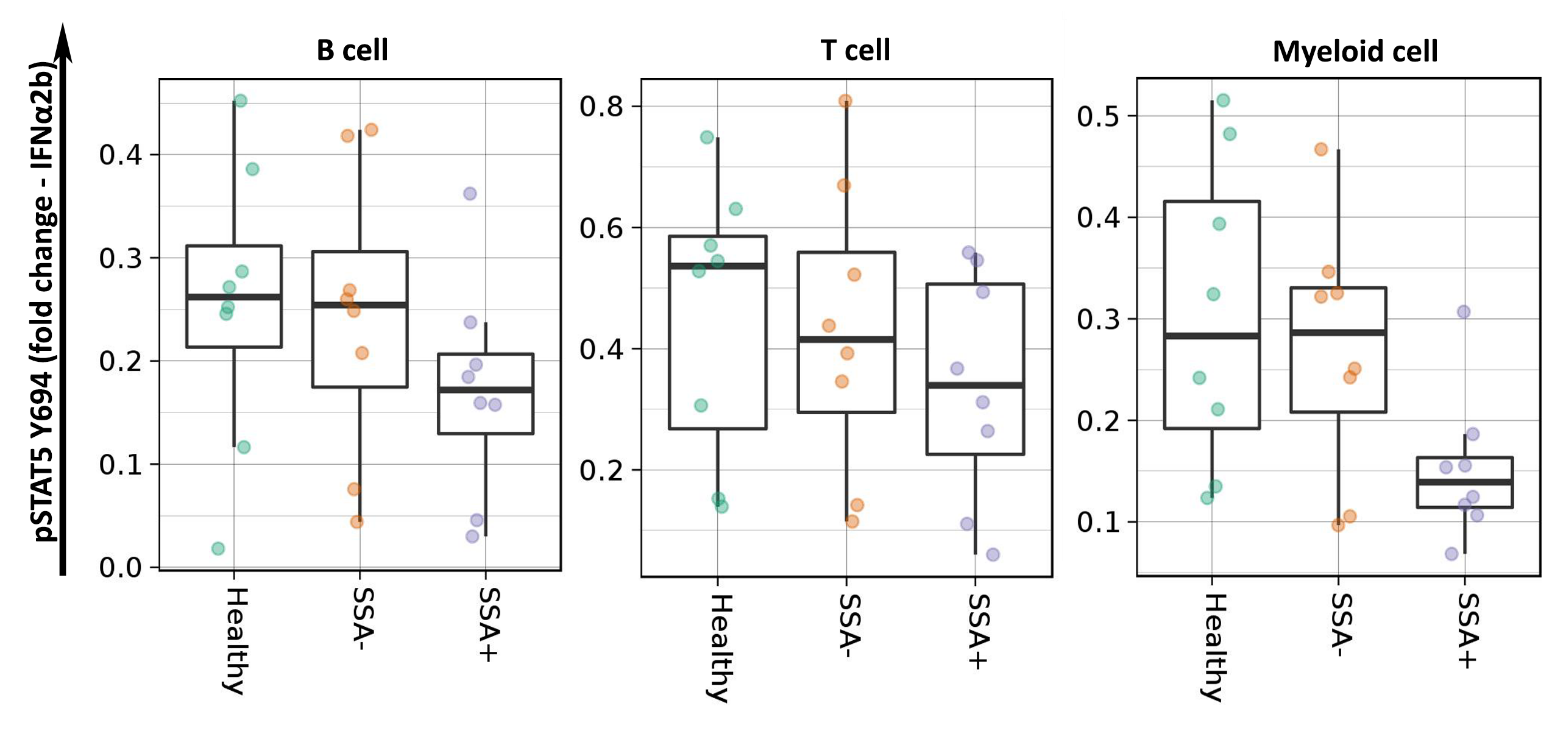
**

**Figure S8. Discrepancies in pSTAT5 Y694 signaling in the parent populations of PBMC in SSA- and SSA+ pSS patients compared to healthy controls, upon IFNα2b stimulation.** Assessment of the level of pSTAT5 Y694 induction by IFNα2b stimulation was done by mass cytometry. Comparisons were made among healthy controls/Healthy (n=8, green circles), SSA- pSS patients (n=8, orange circles) and SSA+ pSS patients (n=8, purple circles). Differential expressions were analyzed using the limma R package and are expressed in terms of fold change. Median fold changes are indicated for each box and values were considered statistically significant for FDR/adjusted p < 0.05.

**
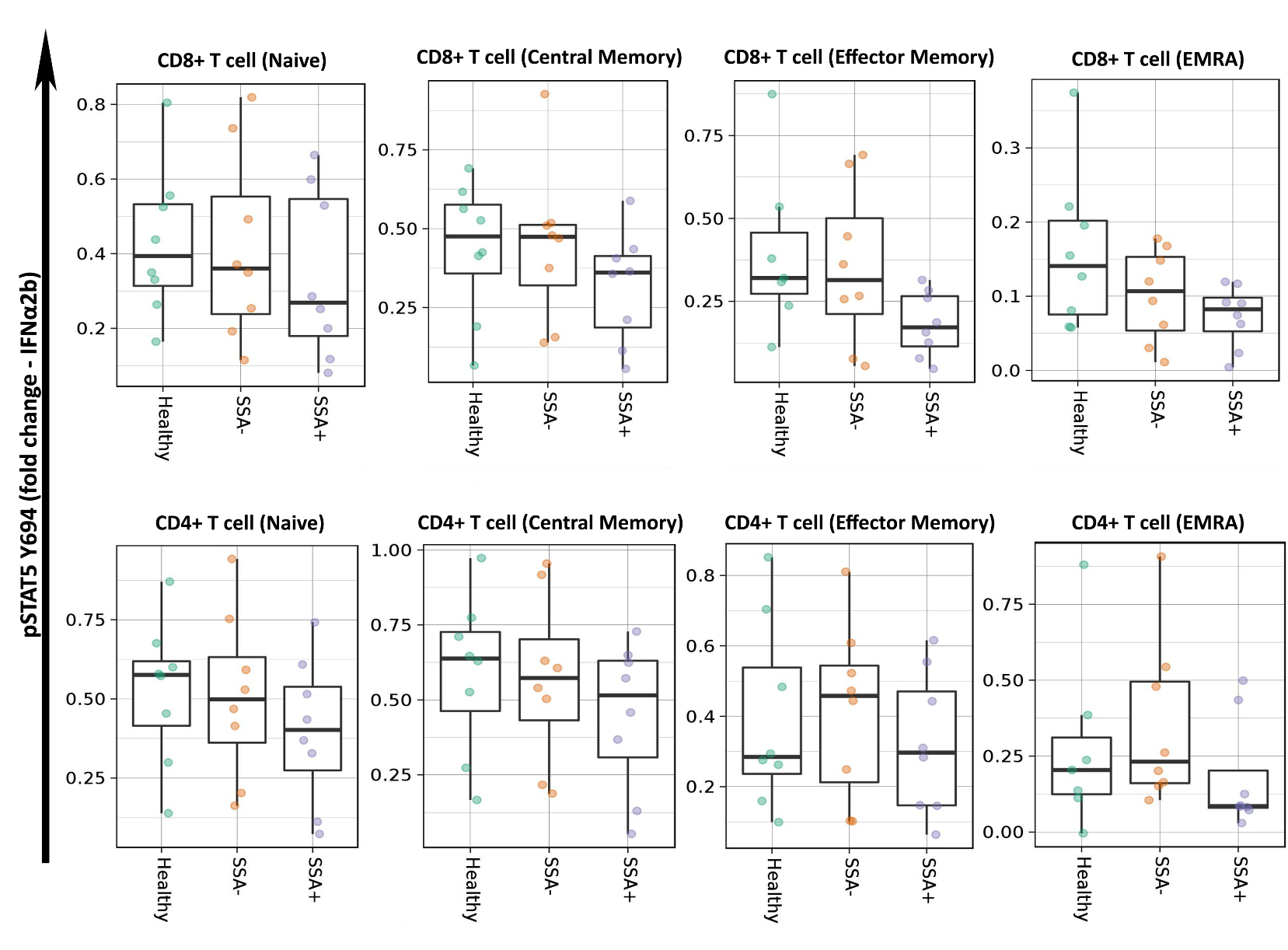
**

**Figure S9. Discrepancies in pSTAT5 Y694 signaling in the T cell sub-populations in SSA- and SSA+ pSS patients compared to healthy controls, upon IFNα2b stimulation.** Levels of pSTAT5 Y694 induction upon IFNα2b stimulation were analyzed by mass cytometry. Comparisons were made among healthy controls/Healthy (n=8, green circles), SSA- pSS patients (n=8, orange circles) and SSA+ pSS patients (n=8, purple circles). Differential expressions were analyzed using the limma R package and are expressed in terms of fold change. Median fold changes are indicated for each box and values were considered statistically significant for FDR/adjusted p < 0.05.

**
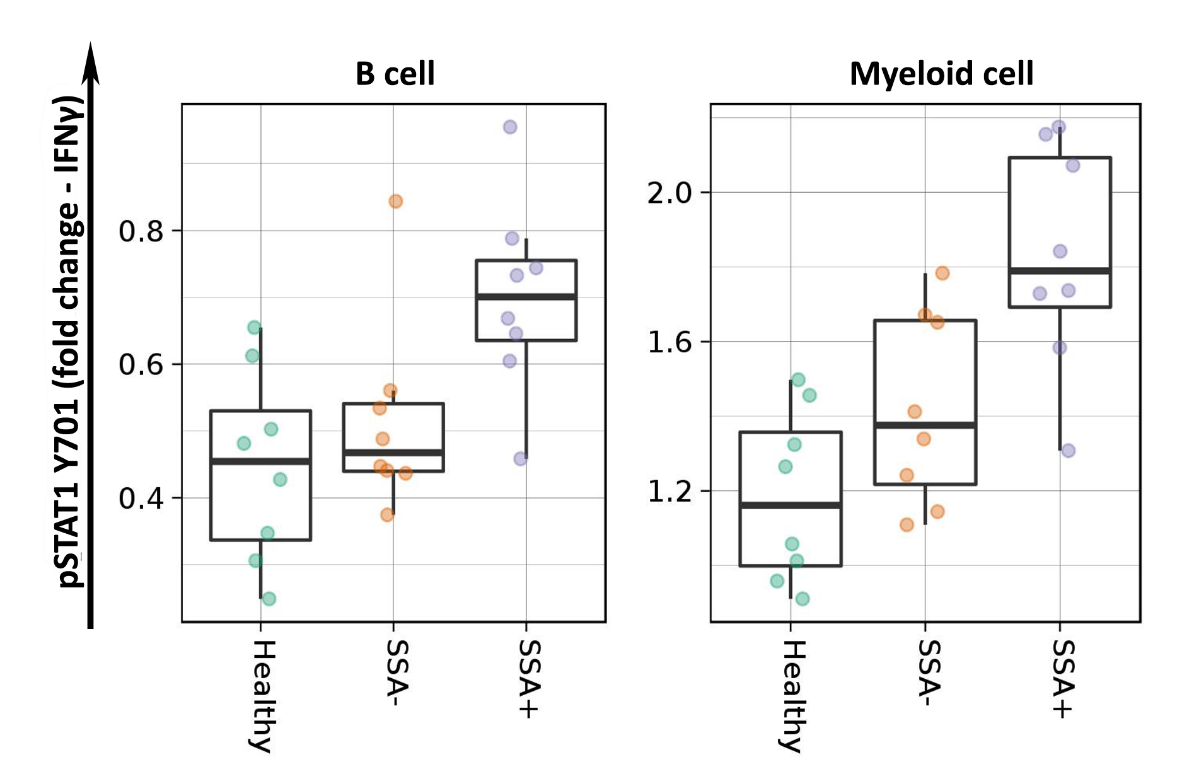
**

**Figure S10. Differential STAT1 Y701 signaling in B cells and myeloid cells of SSA- and SSA+ pSS patients compared to controls, upon IFNγ stimulation.** Levels of STAT1 Y701 phosphorylation in B cells and myeloid cells upon IFNγ stimulation were evaluated by mass cytometry. Comparisons were made among healthy controls/Healthy (n=8, green circles), SSA- pSS patients (n=8, orange circles) and SSA+ pSS patients (n=8, purple circles). Differential expressions were analyzed using the limma R package and are expressed in terms of fold change. Median fold changes are indicated for each box and values were considered statistically significant for FDR/adjusted p < 0.05.

**
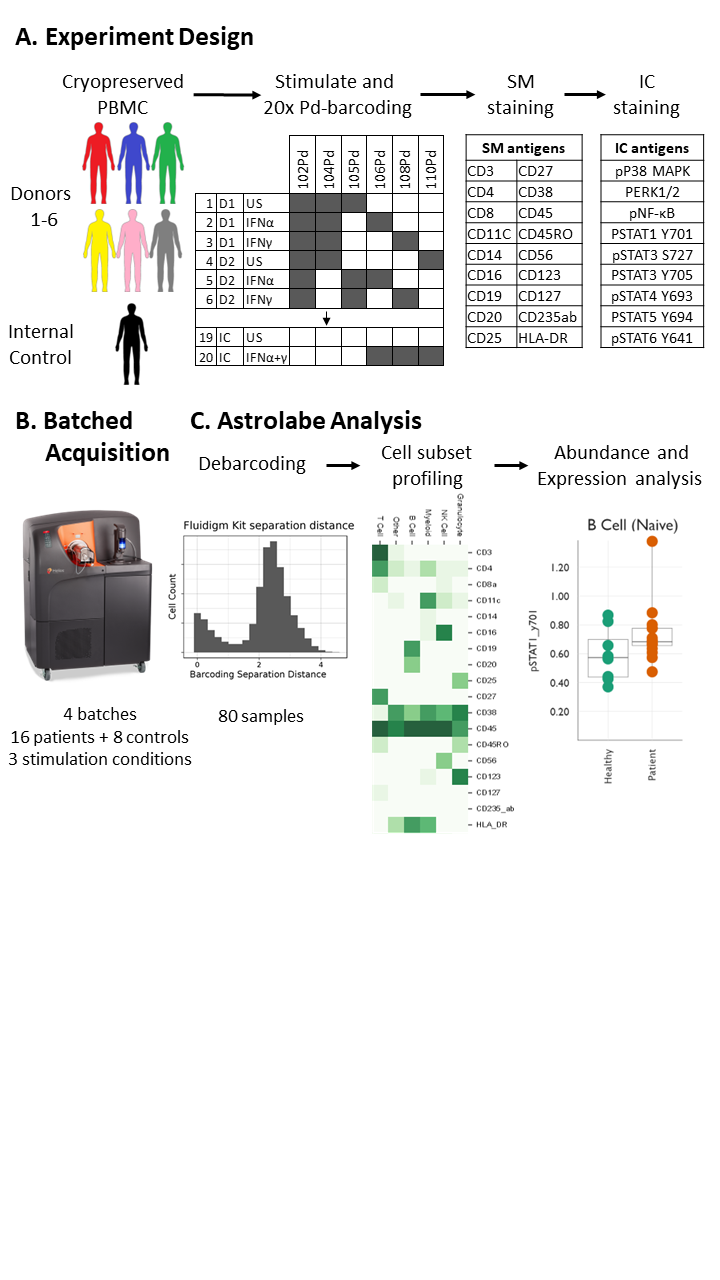
**

**Figure S11. Sample processing and analysis pipeline used in this study.** Peripheral blood from patients (n = 16) and matched healthy donors (n = 8) were drawn, PBMC were isolated and cryopreserved until later date. A. PBMC were thawed and processed in 4 batches of 6 donors, and 1 internal control (IC) which was used in all 4 batches. PBMC were stained with cisplatin for live-dead discrimination, spilt into 3 samples and stimulated with IFNγ, IFNα2b or DMSO (US), with the IC split into 2 samples (1x DMSO, 1x IFNγ + IFNα2b). Following samples were fixed, transiently permeabilized and barcoded with a unique combination of palladium isotopes and pooled into a single sample. The sample was then stained with antibodies to extracellular epitopes (SM staining, see table 2). Permeabilized with methanol and stained with antibodies to intracellular epitopes (IC staining, see table 2) before fixation and staining with iridium (IR) followed by cryopreservation at -80C. B. Following PBMC preparation, cryopreserved barcoded PBMC sample pools were thawed and acquired on a HELIOS CYTOF system with at least 4x10^6^ events measured per barcode with 2x10^5^ events per sample on average. The resulting FCS files were normalized to EQ beads. C. Normalized FCS files were uploaded to astrolabe analysis platform, each batch was debarcoded, identifying individual samples and cell stimulation conditions. Cell subset profiling was utilized to cluster similar cell types based on marker expression and align cluster identity with known cell subsets. Cell subset abundance and antigen expression levels for each cell subset were then compared across donor features (Disease status, SSA autoantibodies).

**
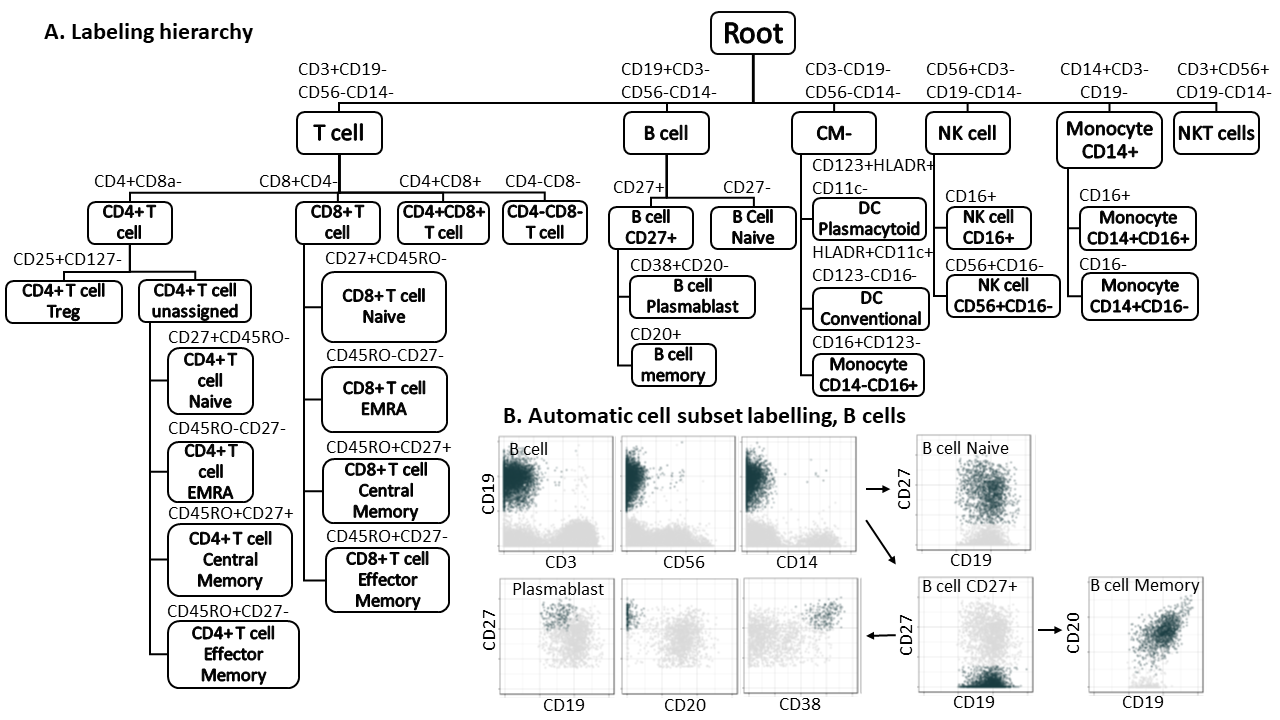
**

**Figure S12. A. Labeling hierarchy generated by the Astrolabe cytometry platform.** First, events were clustered and automatically labeled following a manually curated gating hierarchy. ‘Root’ represents all CD45+ cell events. It denotes the beginning of the cell labelling process, and all cell subsets branch out from it. ‘CM-’ corresponds to negative canonical markers This means that these cells are negative for all the canonical markers that were defined in the Root level i.e., CD3-CD14-CD19-CD56-. ‘Unassigned’ denotes all cells that do not belong to any other subsets under its parent. B. An example for a single sample of the automatic subset identification for B cell branch is given, first B cells are identified based on their expression of canonical markers (CD19+, CD3-, CD56-, CD14-) as indicated by the dark points. CD27+ and CD27- (Naïve B cells) are then identified, with CD27+ B cells further differentiated as memory B cells based on CD20+ expression, and plasmablasts based on positive expression of CD27 and CD38, and a lack of CD20 expression.

**
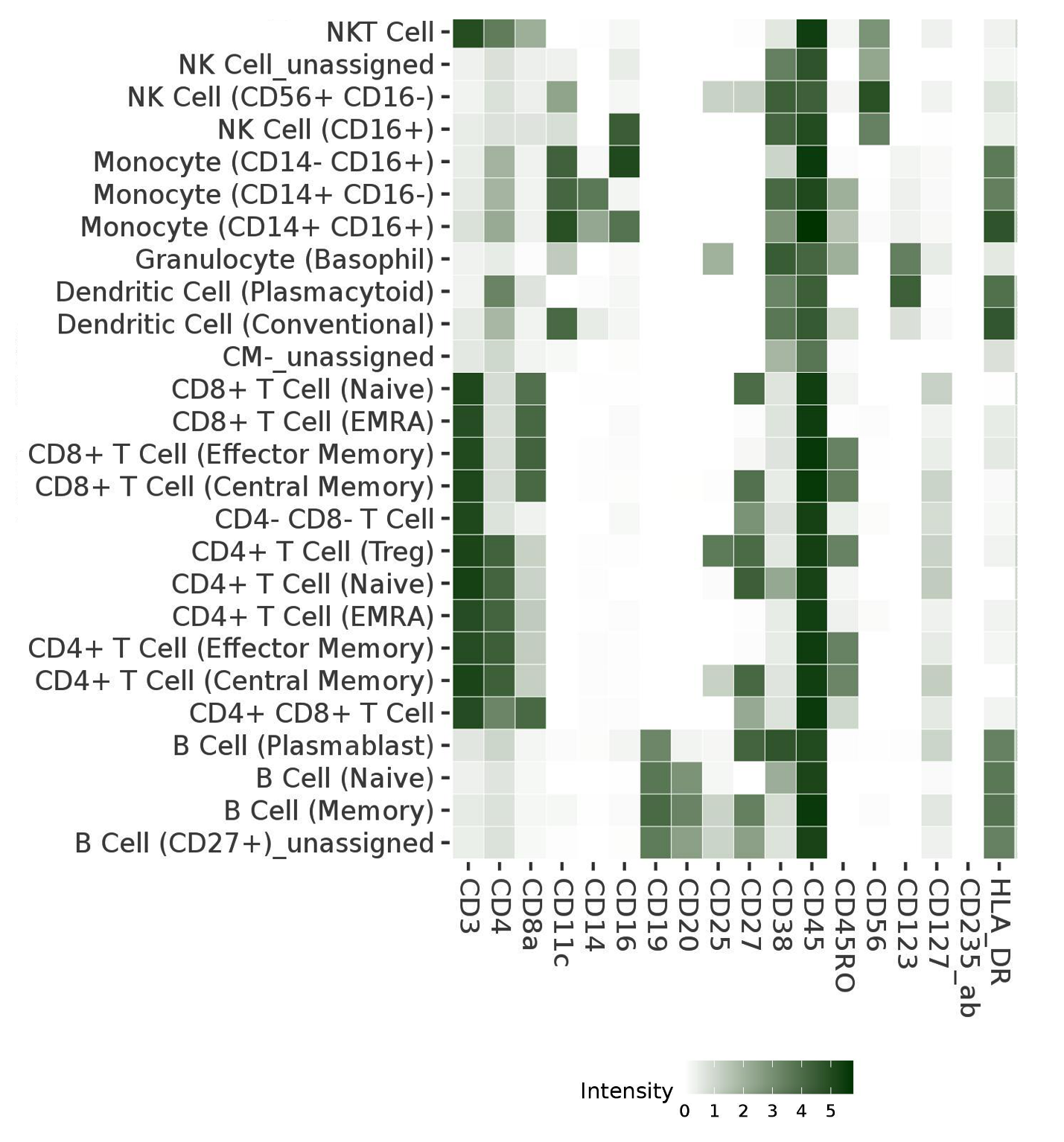
**

**Figure S13. Heatmap of the expression of the surface markers in all the cell types from one sample.** The expression of the surface markers in all the identified cell subsets from one sample are shown. The colour gradient indicates median intensity of expression. Heatmap generation was done using the Astrolabe Cytometry Platform.
